# The risk of type 2 oral polio vaccine use in post-cessation outbreak response

**DOI:** 10.1186/s12916-017-0937-y

**Published:** 2017-10-04

**Authors:** Kevin A. McCarthy, Guillaume Chabot-Couture, Michael Famulare, Hil M. Lyons, Laina D. Mercer

**Affiliations:** Institute for Disease Modeling, Bellevue, WA USA

**Keywords:** Poliovirus, Eradication, Oral polio vaccine, Cessation, Vaccine-derived poliovirus

## Abstract

**Background:**

Wild type 2 poliovirus was last observed in 1999. The Sabin-strain oral polio vaccine type 2 (OPV2) was critical to eradication, but it is known to revert to a neurovirulent phenotype, causing vaccine-associated paralytic poliomyelitis. OPV2 is also transmissible and can establish circulating lineages, called circulating vaccine-derived polioviruses (cVDPVs), which can also cause paralytic outbreaks. Thus, in April 2016, OPV2 was removed from immunization activities worldwide. Interrupting transmission of cVDPV2 lineages that survive cessation will require OPV2 in outbreak response, which risks seeding new cVDPVs. This potential cascade of outbreak responses seeding VDPVs, necessitating further outbreak responses, presents a critical risk to the OPV2 cessation effort.

**Methods:**

The EMOD individual-based disease transmission model was used to investigate OPV2 use in outbreak response post-cessation in West African populations. A hypothetical outbreak response in northwest Nigeria is modeled, and a cVDPV2 lineage is considered established if the Sabin strain escapes the response region and continues circulating 9 months post-response. The probability of this event was investigated in a variety of possible scenarios.

**Results:**

Under a broad range of scenarios, the probability that widespread OPV2 use in outbreak response (~2 million doses) establishes new cVDPV2 lineages in this model may exceed 50% as soon as 18 months or as late as 4 years post-cessation.

**Conclusions:**

The risk of a cycle in which outbreak responses seed new cVDPV2 lineages suggests that OPV2 use should be managed carefully as time from cessation increases. It is unclear whether this risk can be mitigated in the long term, as mucosal immunity against type 2 poliovirus declines globally. Therefore, current programmatic strategies should aim to minimize the possibility that continued OPV2 use will be necessary in future years: conducting rapid and aggressive outbreak responses where cVDPV2 lineages are discovered, maintaining high-quality surveillance in all high-risk settings, strengthening the use of the inactivated polio vaccine as a booster in the OPV2-exposed and in routine immunization, and gaining access to currently inaccessible areas of the world to conduct surveillance.

**Electronic supplementary material:**

The online version of this article (10.1186/s12916-017-0937-y) contains supplementary material, which is available to authorized users.

## Background

April 2016 marked the global cessation of the use of the Sabin-strain oral polio vaccine type two (OPV2) in routine and campaign immunization, with all 155 OPV-using countries switching from the trivalent to the bivalent form of OPV, which contains only vaccine types 1 and 3 [1]. The last case of naturally occurring wild type 2 poliovirus (WPV2) was observed in India, in 1999 [2]. OPV2 is a live, attenuated virus, capable of genetic reversion to a neurovirulent phenotype that imposes a health burden due to vaccine-associated paralytic polio (VAPP). The Sabin-strain viruses are also capable of transmission, and in low-immunity settings can establish circulation; these established lineages are termed circulating vaccine-derived polioviruses (cVDPVs) [3–6]. Among the three OPV serotypes, OPV2 is estimated to cause 40% of all VAPP cases and 90% of all cVDPV cases [7]. The successful removal of OPV2 from elective use therefore presents clear public health benefits. However, the cessation of OPV2 immunization carries the implicit risk that Sabin-strain lineages will survive to become cVDPV2s in the future, necessitating outbreak response with OPV2, and thereby potentially seeding new lineages [8, 9]. Historical experience has established that Sabin-strain polioviruses (and other live, attenuated polioviruses) are able to broadly circulate within populations when introduced after relatively brief (1–3 year) interruptions in OPV use [10–13]. The possibility of a cycle in which OPV2 use in outbreak response seeds new cVDPV2 lineages, necessitating further outbreak response, represents a fundamental risk to the cessation of OPV2 immunization.

Several criteria determine the success of post-cessation outbreak response activities: the response must cover the spatial extent of the (largely unobserved) cVDPV2 transmission, it must achieve sufficient coverage to interrupt transmission within the response region, and the Sabin 2 deployed in the response must not generate new cVDPV2 lineages. This manuscript addresses the conditions under which OPV2 use in outbreak response could establish new chains of Sabin 2 transmission, and how this risk evolves as population immunity declines post-cessation. The manuscript proceeds with the following topics: a description of the model employed, utilization of the model to investigate the risk of OPV2 use in a variety of scenarios, a discussion of the findings with related policy implications, and conclusions.

## Methods

### Model specification

The generic disease branch of the individual-based disease modeling software EMOD DTK v2.8 was used to model polio transmission [14]; a complete specification of the employed model can be found in the (Additional file 1). Transmission takes place on a network of populations representing Level One administrative divisions (provinces or states) throughout 16 countries in West Africa (details in Additional file 1). Within a province, disease transmission dynamics are governed by a susceptible-exposed-infectious-susceptible equation system with partial immunity, and transmission between the provinces proceeds through individual-level migration. As ~ 98% of all cVDPV2 paralysis cases in the AFRO region have arisen in the cohort of children under 5 years of age (polio paralysis data from the Polio Information System (POLIS)), the model tracks only infection and transmission in the under-5 cohort.

### Modeling scenarios

Many factors affect an outbreak response activity’s propensity to establish new VDPV2 lineages: population intestinal immunity at the time of outbreak response, the base reproductive rate *R*_0_ of the Sabin type 2 virus (which may change during genetic reversion), and the epidemiological connectedness of spatially separated populations. Each of these factors is also highly uncertain and varies with the geographical/societal context under consideration. In this work, a variety of potential parameter scenarios are considered (see Table 1), and in each scenario, 1000 iterations of the model are run to quantify the risk that an outbreak response conducted according to existing protocol will seed a new VDPV2, as a function of the time since cessation and the mean per-person, per-day migration rate between provinces.

**Table 1:** Description of parameters varied in the simulation scenarios

Quantity varied	Values
Final base reproductive rate of reverted VDPV2 (*R*_0f_)	{1.2, 1.5, 2.0, 3.0}
Initial reproductive rate of OPV2 as fraction of final *R*_0_ (*g*)	{0.25, 0.5}
Exponential timescale of *R*_0_ reversion (λ)	{60 days, 150 days}
No. of inactivated polio vaccine doses given to children born after OPV2 cessation (*N*_IPV_)	{0, 1}
Distance dependence of migration rates (*c*)	{–1, –2} (1/*d*, 1/*d* ^2^)
Population intestinal immunity in cohort of children born before OPV2 cessation	Induced by three OPV campaigns at 80% coverage and 50% take (moderate immunity) or three OPV campaigns at 100% coverage and 100% take (high immunity)

For simplicity, initial population intestinal immunity^1^ is treated as constant across the provinces. The cohort of children old enough to have been alive at cessation is initialized with one of two immunity profiles: one consistent with having experienced three rounds of OPV2 distribution at 80% population coverage (independent coverage per round) and 50% vaccine take (that is, 50% successful intestinal immunization in fully susceptible recipients), and a second profile with three rounds at 100% coverage and 100% take (an unrealistic assumption but useful for comparison). The cohort born since cessation is assumed to be OPV2-naïve, but depending on the scenario, they may receive zero or one dose of the inactivated polio vaccine (IPV), which induces strong protection from paralysis (humoral immunity), little protection against acquisition and onward transmission (intestinal mucosal immunity) in OPV2-naïve individuals, and a strong intestinal mucosal boosting response in OPV2-exposed individuals (details in Additional file 1) [15–19]. No waning of intestinal immunity over time is modeled.

The survival of VDPV2 lineages from pre-cessation OPV2 use is not modeled here, though the mechanism of emergence and survival would also depend on some of the unknowns studied here; it is simply assumed that an outbreak response has been triggered at a given time since cessation. This approach limits this work from addressing other critical features of VDPV2 control after cessation — the probability of cVDPV2 survival, its ability to remain undetected, and its spatial extent at detection are all key phenomena of interest, and each depends on the infectivity, immunity, and migration parameters varied in the scenario analyses presented below. This work focuses solely on addressing the future cVDPV2 risk associated directly with the outbreak response itself. The recent discovery in Borno State, Nigeria of cVDPV2 and WPV1 viruses from lineages unobserved for 2 and 5 years, respectively, demonstrates that prolonged unobserved circulation is feasible under suboptimal surveillance [8, 20, 21]. In the model, an initial rapid-response OPV2 campaign targets Zamfara State, Nigeria. Sixteen days later, an OPV2 campaign targets Zamfara and the bordering states Sokoto, Katsina, Kaduna, and Kebbi, followed by a joint OPV2/IPV campaign (taking advantage of IPV’s mucosal boosting effect in OPV-exposed individuals) and a third OPV2 campaign in the same states at 4-week intervals.

**Fig. 1:**
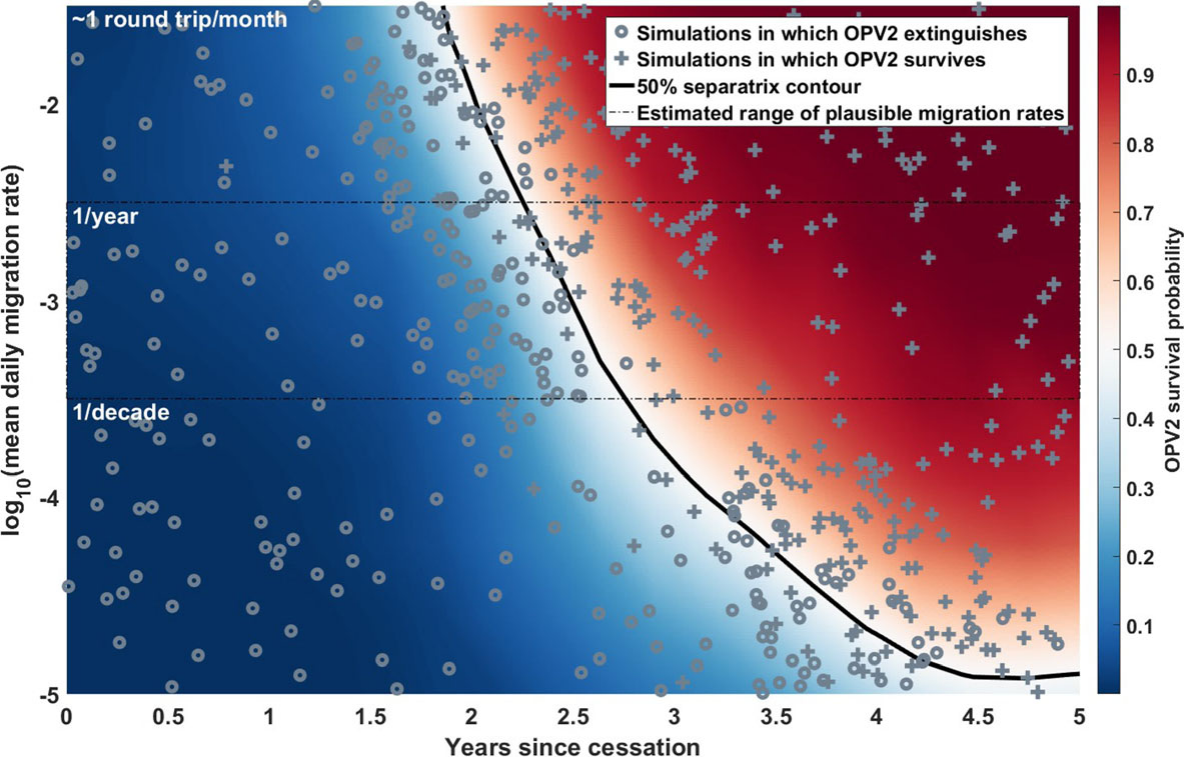
Example output from a single separatrix scenario, with *R*_0f_ = 2.0, *g* = 0.5, λ = 60 days, *N*_IPV_ = 1, *c* = 1. The *colored surface* represents the inferred probability that the OPV2 used in outbreak response continues to circulate, outside of the response region, 9 months after the final response campaign; in *blue regions*, the OPV2 deployed in outbreak response is likely to fail to establish long-term circulation, and in the *red regions*, the OPV is more likely to successfully export from the response region and survive for 9 months in simulation. The *black solid line* represents the parameter contour along which this survival probability is 50%. *Gray crosses* represent individual simulations in which this exportation and survival outcome occurs, and *gray circles* represent those in which it does not. The *thin black dashed box* indicates migration rates that are preferred by a calibration to a single traveling WPV1 outbreak in the region, in 2008. The *y*-axis, mean daily migration rate, is the average rate at which any simulated individual leaves their home province to visit another province; all migration is round trip with a mean trip duration of 1 day. The distribution of simulated points illustrates the behavior of the algorithm; the first round of the Separatrix algorithm broadly explores the space, and the second concentrates simulations around the contour of interest

The infectious dynamics of the different provinces are connected by individuals migrating (in short-duration round trips) between provinces. Each migration is assumed to follow a gravity model, in which the per-day rate of an individual taking a round trip from province *i* to c of the form:

$$ {M}_{ij}=\kappa \frac{\ {p}_j}{d_{ij}^c} $$

where *M*_*ij*_ is the per-person, per-day rate of travel from province *i* to *j*, *p*_*j*_ is the population of the destination province *j*, *d*_*ij*_ is the distance between the population-weighted centers of provinces *i* and *j*, and *K* is a parameter that scales the total migration rate from all sources to all destinations. As *M*_*ij*_ represents a per-person, per-day rate, a source population term (*p*_*i*_) that usually appears in the formulation of a gravity model is implied here. Two values of the exponent *c* in this equation are explored, *c* = 1 and *c* = 2. Setting *c* = 1 results in comparatively more long-distance migration to population centers, and *c* = 2 results in comparatively more migration to nearby provinces. The overall probabilities are scaled up and down by varying the value of *K*. In Figs. 1, 3, 4, and 5, the *y*-axis represents varying values of *K*, translated onto a scale representing the mean per-day migration probability of all people in the simulation for easier interpretation.

While the recovery of neurovirulence during genetic reversion from Sabin is largely understood, with major and minor attenuating sites identified, it is less clear how (or whether) the transmissibility of Sabin virus changes during genetic divergence from the reference Sabin strain [22, 23]. Therefore, instead of utilizing a detailed viral evolution model, here we assume that the infectivity of Sabin 2 virus is some fraction *g* of the fully reverted infectivity, and that it follows an exponential approach to a final infectivity (Additional file 1 Eq. 2). We specifically set *g* to 0.25 or 0.5 in the modeling scenarios and also vary the values of the final infectivity and the timescale of the exponential approach as described in Table 1.

### Post-cessation simulations

A Separatrix algorithm [24] is used to explore the risk of OPV2 survival in the outbreak response described above, as a function of the time since cessation and the mean per-person, per-day migration rate. This algorithm is an iterative approach to inferring the probability of a binary yes/no outcome from a model that is subject to both stochastic uncertainty and parameter uncertainty, and particularly aiming to find the set of points in model parameter space where this probability is equal to some user-defined value. In this case, the outcome of interest is whether or not the OPV2 used in the modeled vaccination response generates a long-lived circulating lineage, the parameter space is the time since cessation and the mean per-person, per-day migration rate, and the user-defined threshold is 50%. A new circulating lineage is considered to have arisen whenever there are individuals infected with the virus, outside of the original response region, 9 months after the outbreak response.

Each combination of the parameters in Table 1 represents a single scenario. For each scenario, the Separatrix algorithm first selects 500 pairs of points in the two-dimensional (2D) space of time since cessation and migration rate, and the model is run with these inputs. The probability of OPV2 survival is estimated throughout the 2D space, and 500 new points are selected to specifically gain more information about the contour in parameter space at which the probability of OPV2 survival is 50%. The algorithm then terminates, rather than continuing to iterate.

## Results

**Fig. 2:**
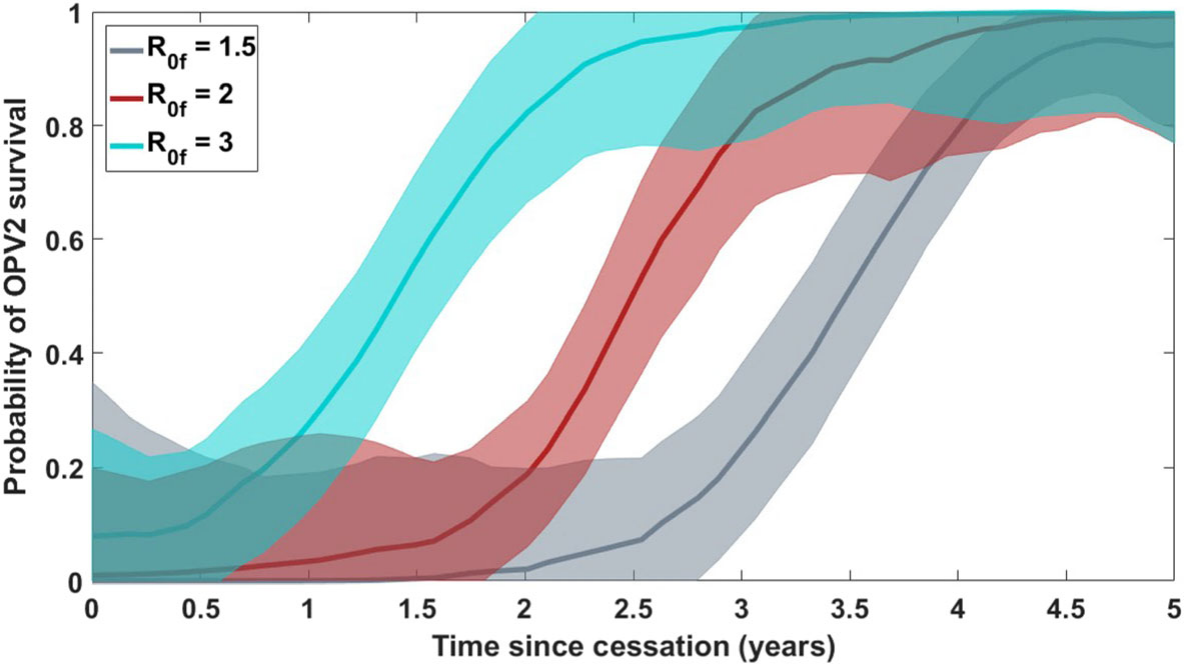
Estimated probability with uncertainty of OPV2 survival for *R*_0f_ = 1.5 (*gray*), 2.0 (*red*), and 3.0 (*cyan*), vs. the time since cessation, at a fixed value of 0.001 for the mean per-person, per-day migration rate (or *y* = –3 on the log-space *y*-axes in Figs. 1, 3, 4, and 5). The *red line* and corresponding uncertainty band correspond to the estimated probability of OPV2 survival along a slice at *y* = –3 through the colored separatrix surface presented in Fig. 1; the *cyan* and *gray* areas represent the same quantity for simulated scenarios run with different values of the final VDPV infectivity. The other scenario parameters are set to *g* = 0.5, λ = 60 days, *N*_IPV_ = 1, *c* = 1. The uncertainty bands represent uncertainty on the estimated probability of the OPV2 survival outcome at a given point in parameter space and do not incorporate uncertainty in the simulation input parameters themselves or other extrinsic sources of uncertainty

**Fig. 3:**
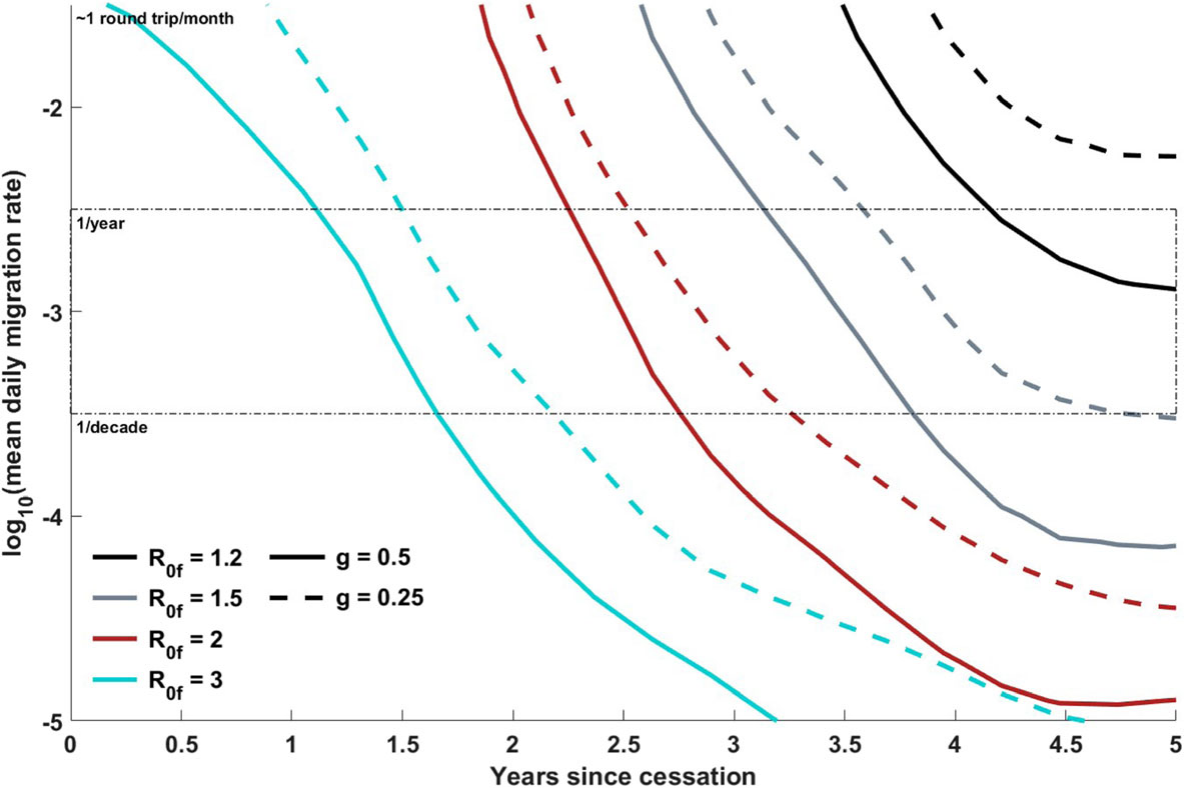
Position of the 50% separatrix line as the *R*_0_ profile of OPV2 varies, at constant λ = 60 days, *N*_IPV_ = 1, *c* = 1. Each separatrix line, for a given scenario, divides the region of parameter space in which OPV2 survival is estimated to be < 50% probable from the region in which OPV2 survival is estimated to be > 50% probable. The “less probable” region is always the region left and below the separatrix line (that is, less connectivity or less time since cessation reduces the probability of survival), and the “more probable” region for a given scenario is above and to the right of the corresponding separatrix line. The *solid* and *dashed lines*, respectively, indicate *g* = 0.5 and *g* = 0.25, while the *cyan*, *red*, *gray*, and *black lines*, respectively, indicate *R*_0f_ values of 3, 2, 1.5, and 1.2. The *thin black dashed box* indicates migration rates that are preferred by a calibration to a single traveling WPV1 outbreak in the region, in 2008. The final *R*_0_ is observed to have the dominant effect, with the risk at a given time point and migration rate decreasing with *R*_0f_ as expected. The initial *R*_0_ multiplier has a comparatively small effect, but a lower initial *R*_0_ does also mitigate the survival risk

**Fig. 4:**
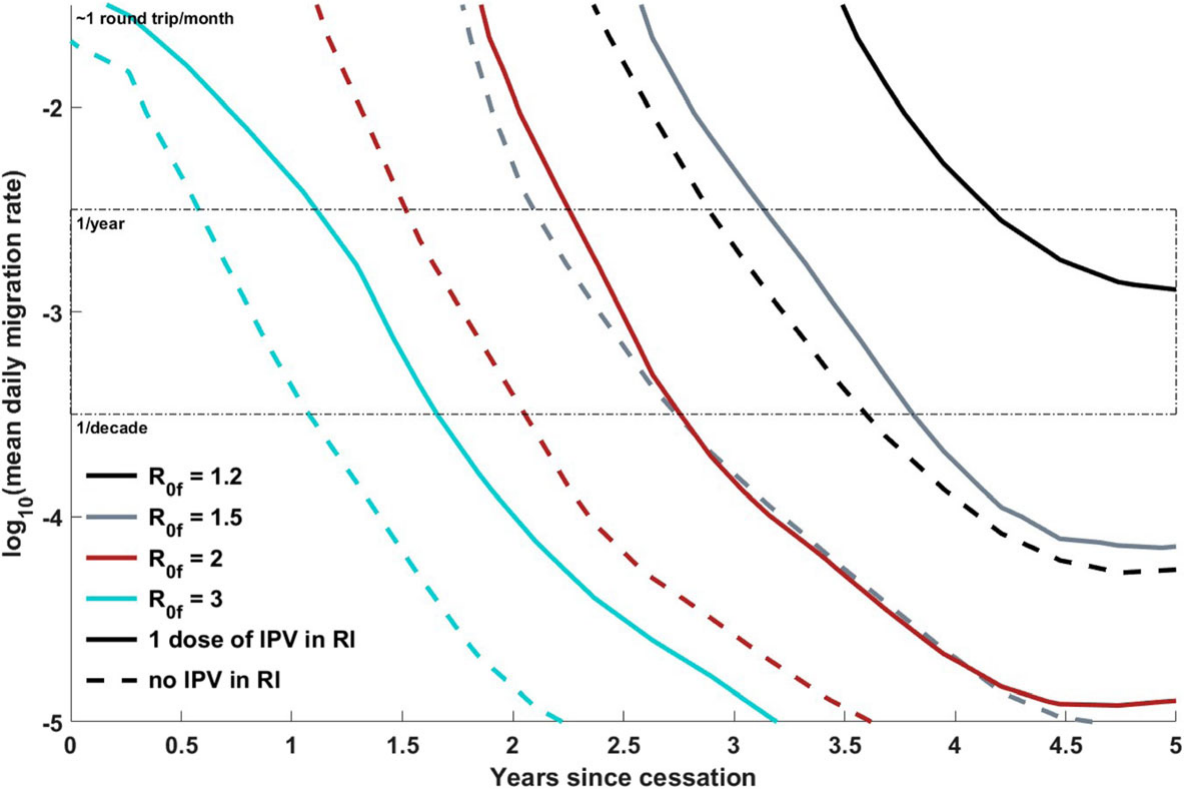
Position of the 50% separatrix line as number of IPV doses in routine immunization varies, at constant λ = 60 days, *g* = 0.5, *c* = 1. The *dashed* and *solid lines*, respectively, indicate *N*_IPV_ = 0 or 1, and the *cyan*, *red*, *gray*, and *black lines*, respectively, indicate *R*_0f_ values of 3, 2, 1.5, and 1.2. The *thin black dashed box* indicates migration rates that are preferred by a calibration to a single traveling WPV1 outbreak in the region, in 2008. Under the assumptions made in this model regarding the population-level effects of IPV dosing, an additional dose of IPV in routine immunization in the cohort born after cessation provides a strong mitigating effect on the risk of OPV2 survival and circulation at low *R*_0_; the mitigating effect declines as the *R*_0_ of the reverted virus increases

Figure 1 presents the output of a single run of the Separatrix algorithm, with *R*_0f_ = 2.0, *g* = 0.5, λ = 60 days, *N*_IPV_ = 1, *c* = 1 (see Table 1 for definitions of the symbols). Figures 1, 2, 3 and 4 all present comparisons at the moderate immunity profile (defined in Table 1) in the pre-cessation birth cohort. The color surface shows the imputed risk throughout a 2D space of mean migration rate and time since cessation; the gray crosses and circles indicate simulations in which a new lineage succeeds or fails, respectively, to establish long-term circulation (defined for the purposes of this study as continued viral transmission, outside of the response provinces, 9 months after the outbreak response); the thin black dashed box outlines a space of migration rates preferred by a calibration to a previous traveling outbreak of WPV1 in the region (Additional file 1); and the black line represents the 50% separatrix line, the imputed contour in parameter space along which the VDPV2 risk is 50%. In this scenario, this line indicates that this risk reaches 50% around 2.5–3.5 years post-cessation, depending on the migration rate.

Figure 2 presents a different look at the mean and 2σ uncertainty intervals on the probability of OPV2 survival vs. time since cessation, along a slice at a fixed migration rate. The gray, red, and cyan curves present results for *R*_0f_ = 1.5, 2.0, and 3.0, respectively, vs. the time since cessation. To relate this to Fig. 1, the red curve directly corresponds to the background color in Fig. 1 along a slice at *y* = –3 (where the average person has a per-day migration probability of 10^–3^), representing the probability of OPV2 survival vs. time and corresponding uncertainty. The other curves present the same quantity from scenarios that differ only in the final VDPV2 *R*_0f_. The other scenario parameters are set to *g* = 0.5, λ = 60 days, *N*_IPV_ = 1, *c* = 1. The bands represent the uncertainty on the estimated probability of the OPV2 survival outcome for a given scenario, at a given point in the parameter space, and do not incorporate any uncertainty in simulation input parameters themselves or other extrinsic sources of uncertainty.

### Scenario comparison: dependence of VDPV2 risk on infectivity profile

It is difficult to visually compare the full risk surfaces of multiple scenarios, so the 50% separatrix contours derived in different scenarios are used to compare the relative risks. Figure 3 illustrates how the risk profile depends on R_0f_ and *g*, with other parameters held constant (λ = 60 days, *N*_IPV_ = 1, *c* = 1). As expected, the risk of continued circulation rises earlier with increasing *R*_0_ of fully reverted OPV2; the lowest tested value, 1.2, presents minimal risk even 5 years post-cessation in the preferred migration rate region, while the highest value, 3.0, presents high risk just 18 months post-cessation. At a given *R*_0f_, changing *g* from 0.5 to 0.25 induces a small but non-negligible shift of the separatrix to later times/higher migration rates. This figure demonstrates that the contexts in which transmission is most intense, and therefore cVDPV2 is most likely to have survived, are also likely to have the shortest windows in which Sabin virus transmission from the response is likely to locally self-extinguish before exporting.

### Scenario comparison: dependence of VDPV2 risk on IPV use in routine immunization post-cessation

Figure 4 illustrates how the inclusion of IPV in routine immunization (RI) affects the risk of OPV2 survival in this model. The coverage of RI is assumed to be 80%. While the herd immunity effects of IPV are evident in developed nations, where the oral-oral transmission route likely dominates [25, 26], they are poorly characterized in developing countries, where the fecal-oral transmission route dominates. At the individual level, recent monovalent OPV2 (mOPV2) challenge studies comparing a variety of mixed IPV-bivalent OPV (bOPV) schedules have found that mixed IPV-bOPV schedules provide heterotypic mucosal immunity to type 2 that appears superior to that from bOPV or IPV alone but inferior to that from mOPV2 or trivalent OPV (tOPV) [16, 17]. It is not immediately apparent from the literature whether additional IPV doses beyond the first induce a dose-dependent increase in this heterotypic immunity, or whether this incremental effect depends on the ordering of bOPV and IPV in the schedule [16, 17]. The observed induced immunity reduces both the probability of acquisition upon mOPV2 challenge and the duration and amount of Sabin 2 shedding in stool. At the population scale, it is unclear how this reduction in acquisition at challenge doses translates to protection at natural exposure levels, and how the reduction in shedding translates to reduced infectiousness in close-contact and community settings in regions of poor sanitation [15, 27]. In this model, it is assumed that children born post-cessation will be bOPV-exposed, and that a dose of IPV in RI will confer some degree of heterotypic protection — a 10% reduction in the recipient’s effective exposure and a 10% reduction in a recipient’s onward infectivity are assumed; given the uncertainties around incremental effects of additional doses, a 2 × IPV RI schedule is not compared here. In the model, the IPV distributed during outbreak response will have similar effects on the OPV2-naïve but will induce a boosting response in the OPV2-exposed. Under these assumptions about bOPV + IPV immunization, Fig. 4 shows that if a dose of IPV in RI does provide small but non-zero reduction in acquisition probability and onward transmission at the individual level, it can substantially mitigate OPV2 survival if the reverted Sabin *R*_0,f_ is low, but this mitigating effect disappears as *R*_0,f_ increases. From a policy perspective, this result rings familiar. High coverage of IPV in RI has managed to eliminate or prevent re-establishment of WPV in populations with high-quality, widespread sanitation and high socioeconomic status, but in contexts amenable to robust transmission of poliovirus (generally contexts in which the coverage of RI is also quite low), IPV’s limited intestinal protection is unlikely to block OPV2 transmission if reintroduced in outbreak response.

### Scenario comparison: dependence of VDPV2 risk on immunity profile at OPV2 cessation

**Fig. 5:**
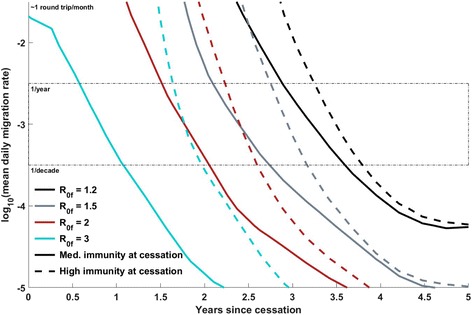
Dependence of the position of the 50% separatrix line on immunity levels in the cohort of children born before cessation: 100% immunity (*dashed lines*) vs. immunity induced by three rounds of OPV at 80% coverage, 50% take (*solid lines*). All lines at constant *g* = 0.5, *N*_IPV_ = 1, *c* = 1, λ = 60 days. The *cyan*, *red*, *gray*, and *black lines*, respectively, indicate *R*_0f_ values of 3, 2, 1.5, and 1.2. The final *R*_0_ is observed to have the dominant effect. The *thin black dashed box* indicates migration rates that are preferred by a calibration to a single traveling WPV1 outbreak in the region, in 2008. The effect of increasing immunity in the older cohort is largest at higher *R*_0_, as higher *R*_0_ facilitates more transmission through partially immune older children. However, the additional protection is somewhat modest (considering the extreme assumption of perfect immunity in all children born pre-cessation), indicating that the cohort of children born post-cessation rapidly becomes a dominant contributor to OPV2 transmission in this model

Finally, Fig. 5 compares the two potential pictures of immunity at the time of cessation. The solid lines indicate simulations with pre-cessation population immunity in children aged 0 to 5 years old induced by three OPV campaigns at 80% coverage, 50% take; the dashed lines indicate simulations with 100% coverage, 100% take (essentially, perfect immunity within this cohort). The dashed lines essentially indicate the time at which the cohort of children born after OPV2 cessation will be able to sustain circulation of OPV2 in the absence of any transmission through the older cohort. The duration of the additional protection from perfect pre-cessation immunity increases as *R*_0,f_ increases, as the virus is increasingly able to recruit the partially immune older children into the transmission chain. The additional protection against cVDPV2 establishment provided by perfect immunity in the older cohort is modest given the extreme nature of this assumption, as the naïve cohort of children born post-cessation eventually grows sufficiently large to sustain transmission.

Other scenarios exploring uncertainty in the distance dependence of the gravity model of migration, the reversion rate of OPV infectivity, and the duration of Sabin 2 persistence result in smaller effects on the risk profile and are presented in the (Additional file 1).

## Discussion

The population immunity conditions in the upcoming years will be unprecedented; little to no immunity will be acquired through natural infection as in the pre-vaccine era, and type 2 immunity will be provided solely through IPV and limited heterotypic effects of bOPV, with little ability to induce strong intestinal mucosal immunity. Any observed cVDPV2 must be extinguished, and OPV2 is the best currently available tool for doing so, but outbreak response activities post-cessation will infect a sizable population with the OPV2 virus in a world with an ever-growing young cohort lacking intestinal mucosal immunity. While uncertainty in immunity, transmission, and migration conditions prevents a strongly constrained estimate of this risk vs. time in a particular context, the results of this study indicate that under a wide range of conditions, outbreak responses as currently outlined could potentially create a cascade of new outbreaks within 18 months to 4 years post-cessation.

In the near term, minimizing the risk of cascading cVDPV2 outbreaks requires strategies that minimize the risk that OPV2 use will be required at all in the future.

1. *Strengthen both paralysis-based and environmental poliovirus surveillance*. In the absence of OPV2 use, the emergence of cVDPV2 relies on the unobserved survival of lineages seeded before cessation. Detecting and interrupting any cVDPV2 lineages currently circulating, while global population immunity is high, minimizes the risk of cascading cVDPV2 outbreaks in the future. Environmental surveillance provides the ability to detect poliovirus in a population in the absence of paralytic cases and could be used to track Sabin 2 survival in the near term to identify places of concern early.
2. *Use aggressive outbreak response in the near term*. The emergence of VDPV2 in the near term will be an effective indicator of locally low population immunity in a world in which immunity remains high. Near-term OPV2 use does not present substantially more risk than did its use immediately pre-cessation. Widespread cVDPV2 circulation has been observed in the past [28, 29], and immunity conditions post-cessation will be unprecedented due to a lack of both natural and vaccine-derived immunity. These facts argue for outbreak responses soon after cessation to be geographically broad, both to ensure interruption of the observed transmission chain and to raise population immunity in regions surrounding the emergence. It may be advantageous to heighten surveillance in neighboring districts or countries known to have imported polioviruses in the past from the emergence region.
3. *Use IPV in immunization campaigns*. A second crucial feature of IPV in the post-cessation world is its ability to boost mucosal immunity against type 2 in OPV2-exposed individuals. While the use of IPV in outbreak response campaigns is already a piece of the outbreak response protocol, IPV immunization campaigns outside of outbreak response could boost type 2 mucosal immunity in the cohort with prior OPV exposure and reduce paralysis burden from cVDPV2 and VAPP in the cohort born post-cessation. This work has not addressed the question of waning mucosal immunity, but if mucosal immunity wanes on relatively short timescales, this waning could be counteracted through IPV boosting, and the value of IPV boosting campaigns targeting older children and adults should be considered by the program.
4. *Obtain access to currently inaccessible areas*. Regions of the world that are currently inaccessible to effective surveillance or outbreak response due to violence, instability, or local resistance present significant risks where VDPV2 lineages could circulate unobserved. In Borno State, Nigeria, many areas have been inaccessible for years due to Boko Haram activity. In this state, both cVDPV2 (environmental isolation March 2016, most recent observed relative from May 2014) and WPV1 (paralysis onsets in July 2016, most recent observed relatives from 2011) have been recently observed [21, 30]. These discoveries highlight the critical risk that inaccessible areas present to the polio eradication and OPV cessation efforts.
5. *Strengthen IPV in routine immunization*. Under the (admittedly uncertain) assumptions about IPV-induced intestinal mucosal immunity used in this study, high-coverage IPV immunization in RI could mitigate the risk of OPV2 survival for a short time. Even if these results overestimate the herd effect of bOPV + IPV cross-protection in the post-cessation cohort, scaling up the coverage and number of doses of IPV in RI would provide valuable individual protection against paralysis, reducing the burden of VDPV2 outbreaks or VAPP caused by OPV2 response.

However, it is important to be mindful of the tension between increasing individual protection against paralysis and reducing the program’s ability to detect cVDPV2 transmission through acute flaccid paralysis (AFP) surveillance, thereby potentially delaying the detection of and response to cVDPV2 lineages. The importance of considering silent circulation has been clearly demonstrated by the WPV1 that circulated in Israel in 2013–2014 and generated no paralysis cases [31, 32], and previous work has considered silent transmission routes through IPV-vaccinated individuals [33] and cohorts with waned intestinal immunity [8]. Other work has found that IPV vaccination should only cause substantial delays in outbreak detection at extremely high coverage levels [34]. Given the example of silent WPV1 circulation in Israel noted above, it is also worth noting that Israel has not reported any cVDPV circulation or emergence, despite switching to IPV in 2004 and being surrounded by OPV-using countries. The technical and ethical complications in trading individual paralysis protection vs. speedier outbreak detection are out of scope for this work, but they should be considered in the broader discussion around IPV’s role in routine immunization in the polio eradication and OPV cessation regime.

Most of these items are already priorities of the Global Polio Eradication Initiative, and the idea that OPV use in a post-cessation world presents a risk of seeding new cVDPVs is not new [9]. These recommendations all emphasize the immediacy of this risk, highlighting that the “honeymoon period,” during which the risks associated with OPV2 use remain low, is transient and could be quite brief. Near-term cVDPV2 outbreak responses must therefore serve the dual purposes of interrupting an observed chain of transmission and preventing the emergence of new ones, and all tools available should be applied during this honeymoon period to minimizing the chances that OPV2 use in outbreak response will become necessary in 2018 or beyond.

The results of this study do not argue against the use of OPV2 in response to cVDPV2 lineages observed in the future, as, absent innovations in polio surveillance or new vaccines, the potential risk of OPV2 response seeding new lineages does not outweigh the known risk of allowing sustained transmission of a neurovirulent strain. OPV2 (in monovalent or trivalent formulation) remains the sole tool demonstrably capable of interrupting type 2 viral transmission in contexts with high transmission intensity and poor sanitation, where cVDPV2 lineages seeded pre-cessation are most likely to survive. Circulating VDPV2 lineages have been discovered and responded to with mOPV2 in six countries in the year between cessation and the writing of this manuscript [35]. Monitoring the survival or disappearance of the Sabin virus in these populations will provide crucial real-world data that should inform the risk of mOPV2 use in the near term. Rather, these results highlight that the relevant timescale depends on assumptions about population immunity, disease transmissibility, and population mobility, but once population immunity becomes low enough to support circulation, it is unclear whether the risks of OPV2 use can be mitigated without new vaccines that induce mucosal immunity without transmitting efficiently or reverting to neurovirulence.

Future work should study the question of whether targeted mOPV2 deployment strategies could mitigate the risk of OPV2 survival while still interrupting circulation of an existing lineage with high probability. Research into vaccines that induce intestinal immunity without transmitting efficiently or acquiring neurovirulence is necessary [36–38], especially as even in the event that all VDPV2 lineages are extinguished, the polio-free world will remain at risk of reintroduction from accidental release, bioterrorism, and long-term poliovirus shedding from immunocompromised individuals [39, 40]. Successful development of such a tool would provide a safer tool for outbreak elimination and immunity maintenance in the post-cessation world.

### Model design choices, limitations, and interpretation

Several design choices and assumptions bear discussion here. As mentioned in the Methods section, the model tracks only infection and transmission in the under-5 cohort, in which the paralytic polio burden in West Africa is concentrated. Because the EMOD DTK is an agent-based model, computational expense grows with the simulated population size, and limiting the model to this age cohort enables simulations of larger effective populations. However, this design assumes that participation of older children in transmission can be integrated into an “effective” transmission rate between under-5 children, and that transmission from older individuals to older individuals without under-5 participation can be neglected. This assumption also limits the model from being able to investigate the effects of mucosal immunity waning at the individual level in the adult population (discussed in, e.g., [8, 41, 42]). Imperfect or waned immunity in the older cohort would raise the risk of OPV2 use generating new cVDPV2 lineages compared to the results of this model.

This individual-based model uses migration of individuals between metapopulations to couple the infectious dynamics. The *y*-axes of Figs. 1, 3, 4, and 5 display a range of per-person, per-year average migration rates used in the model, with a box that outlines the range of migration rates preferred by calibration described in the (Additional file 1). However, real migration is a complex set of behaviors encompassing short-duration round trips, seasonal migration, and long-term relocation of individuals and families, with heterogeneity across age and socioeconomic status. The simple round-trip migration model implemented here does not capture this complexity, and in particular would not capture differential roles of older vs. under-5 children in spatial spread of the disease. However, the use of the same migration model in the calibration exercise and these projections provides a degree of self-consistency, with the understanding that the migration rates are interpreted in this modeling framework as an effective epidemiological coupling between metapopulations.

The evolution of OPV transmissibility is not well constrained by data, as the low case-to-infection ratio of OPV makes the transmission chain difficult to observe. The literature contains several models and reviews that address the ratio of the infectivities of OPV and WPV; 0.15–0.35 [43], 0.56 [44], 0.1–0.2 [41], 0.1 [45] are a few examples. It is also not clear how or whether the transmissibility of OPV evolves as it reverts in the wild, but this is also a feature shared with other models in the literature [44, 45]. These sources guided the choices to set the initial infectivity of Sabin 2 to 0.25 or 0.5 of the eventual infectivity, reached after a few months or a year of reversion (in scenarios with λ = 60 or 150 days, respectively).

The values of *R*_0_ also must be interpreted within the context of the restricted population modeled here — if the model were expanded to include the full population, assuming homogenous mixing and high mucosal immunity in the over-5 cohort, the *R*_0_ values would need to be scaled up to simulate similar dynamics, as modeled infectivity would be diluted into a much larger, higher immunity pool of potential contacts. Specifically, because the under-5 cohort represents about 17% of the total population of the countries modeled [46], the *R*_0_ values should be scaled up by a factor of about 6 for comparison with a full-population model, so the final *R*_0_ values of 1.2, 1.5, 2, and 3 in this model would correspond to about 7.2, 9, 12, and 18 in a full-population, homogenously mixed model (with initial infectivity values scaled as described above). Estimates of *R*_0_ (of OPV or WPV) from other models or past outbreaks, with varying assumptions about heterogeneity in mixing, for comparison, can be found in [8, 22, 33, 44, 47–49], and the range of values considered here (after appropriate rescaling) is in line with values explored in these works.

## Conclusions

As population immunity to type 2 poliovirus transmission declines in upcoming years, the use of OPV2 in outbreak response will present an increasing risk of seeding new cVDPV2 lineages, putting the entire cessation effort at risk. While exact transmission conditions are uncertain and vary across geographic contexts, and the probability of observing new VDPV lineages from pre-cessation OPV use should decline over time, the risk of mOPV2 use if required may grow to alarming levels within as little as 18 months. Without new tools to induce strong mucosal immunity, it is unclear whether this risk can be mitigated in the long term. In the short term, this potential outcome implies a need for strategies that minimize the risk that OPV2 use will be needed in the future: maintaining high-quality surveillance systems, broadening near-term outbreak responses, strengthening access to IPV in routine immunization, and negotiating access to currently inaccessible areas. In the long term, continuing the push for new polio vaccines that can induce mucosal immunity with reduced risks of transmission or reversion is important in the event of accidental or intentional type 2 poliovirus release into a highly susceptible population.

## Additional file

Additional file 1: Supplemental Materials. (ZIP 2.67 mb)

## Abbreviations

bOPV: Bivalent oral polio vaccine
cVDPV2: Circulating vaccine-derived poliovirus type 2
IPV: Inactivated polio vaccine
OPV: Oral polio vaccine
RI: Routine immunization
SIA: Supplemental immunization activity
tOPV: Trivalent oral polio vaccine
VAPP: Vaccine-associated paralytic polio
VDPV2: Vaccine-derived poliovirus type 2
WPV2: Wild poliovirus type 2

## Footnotes

1 Throughout this manuscript, unless otherwise specified, “immunity” will generally refer specifically to intestinal immunity, which protects against viral acquisition and fecal shedding, rather than humoral immunity, which is induced by the inactivated polio vaccine and protective against paralysis, but provides limited at best protection against acquisition and fecal shedding in individuals who have never experienced a live poliovirus infection, either from OPV or WPV.
