# Supplementary material for: The risk of type 2 oral polio vaccine use in post-cessation outbreak response
Source: BMC Med. 2017 Oct 4;15:175. doi: 10.1186/s12916-017-0937-y (PMC5627419; doi:10.1186/s12916-017-0937-y)
Supplement: Additional file 1: — Supplemental Materials. (ZIP 2.67 mb) [file 12916_2017_937_MOESM1_ESM.zip › KM_PostCessation_OPV_Supplement.docx]

**Model Specification**

The EMOD model is an individual-based stochastic model of disease transmission, with support for campaign implementation, heterogeneous transmission, and spatially segregated populations coupled by individual-level migration; the software also includes sub-packages implementing “generic”, vector-borne, water-borne, airborne, and sexual disease transmission. The EMOD software is available for download at <http://idmod.org/idmdoc/#EMOD/EMODBuildAndRegression/BuildingEMODTOC.htm>. The model presented in this manuscript was developed within the EMOD “generic” simulation framework, implementing a discrete-time, individual-based form of a susceptible-exposed-infectious-susceptible model. Below, we review the adjustments to a simple SEIS model that were implemented in this model to reflect polio immunology and specify the model in terms of the individual-level state transition dynamics. Please see Table 1 at the end of this section for a summary of an important subset of parameters employed in the model specification equations, with descriptions, corresponding numerical values or set of values, and references to other models for comparison. A second table is provided in a separate supplemental file and presents an exhaustive list of all symbols used in the coming equations.

Individuals in the model exist in susceptible, exposed, and infectious states, with individuals returning to the susceptible state immediately upon clearance of an infection. However, infections with wild or Sabin-strain attenuated poliovirus, as well as vaccination with the inactivated polio vaccine, are known to confer partial immunity to individuals. Immunity in this model is characterized by two individual-level properties: acquisition-moderating immunity (**α**), which modulates the probability of infection given exposure to infectivity, and applies in the susceptible state; and transmission-moderating immunity (**τ**), which reduces the infectivity of an infectious individual. Both **α** and **τ** are defined on [0, 1], and are defined such that a completely naïve individual has both properties equal to 1. At **α**=0, an individual cannot be infected by any amount of exposure; at **τ**=0, an individual who becomes infected will not shed.

The parameters governing individual immunity are updated upon clearance of OPV infection or successful IPV vaccination. In the event of multiple vaccinations or infections, the **α** and **τ** parameters stack multiplicatively; these parameters thus aim to model a per-infection effect on acquisition- and transmission-modulating immunity. An increased immune response to secondary live infections is a common feature of other models and data reviews in the literature, see [1–5]; while these do not necessarily support the exact multiplicative stacking used in this model, the resulting acquisition and transmission modifiers roughly match those presented in [1], for a single vs. multiple live infections. IPV vaccination behaves similarly, with per-dose effects on the two immunity channels, but IPV can provide two sets of effects; for individuals without a history of OPV infection, an IPV vaccination provides an individual with a small priming effect in each immunity channel, but for individuals with a history of OPV infection, IPV provides a larger boosting effect on immunity. As noted in the main text, current support for an incremental effect of additional IPV doses on the mucosal immunity of bOPV-exposed, OPV2-naïve children is not immediately apparent from the published literature. One recent study supports a dose-dependent effect while another finds no difference, though the two studies are in different settings and use different schedules.[6, 7] This model does in fact implement a dose-dependent response, as it is simpler to implement and it should not substantially affect the results - only a single dose of IPV in RI is modeled and compared against zero doses. A small subset of children in the model may receive the RI IPV, be missed by the subsequent OPV campaigns, but receive a second dose of IPV in outbreak response; this cohort is sufficiently small that its effects are overwhelmed by the uncertainties in other model features (Sabin 2 R_0_, overall demographics of immunity, migration rates and connections).

The infectious and incubation periods ($\gamma_{I}$­ and $\gamma_{E}$, respectively) were modeled using constant times spent in a compartment, rather than the exponential distribution implied by the rate parameters in a standard SEIS model, changing the parameters from rates to delays in the equations. The mortality rate ν is implemented as a function of age ν(*a*), with values obtained from DHS[8]. The model tracks only 0-5 year olds, as ~98% of all cVDPV2 paralysis cases in the AFRO region have arisen in this cohort.[9] As the case load is concentrated in this age group, the authors think it likely that virus transmission is also highly concentrated in this cohort. To the extent that silent circulation occurs in older cohorts, for the purposes of modeling, it is assumed that outbound and inbound transmission from the observable cohort to the silent cohort can be reduced to an effective transmission rate within the observable cohort. The effective birth rate is adjusted to produce a population growth rate of 2.8% per year[8].

The model includes a linear rising exposure to infectiousness with age. This term was used in previous work, modeling WPV1 paralysis cases in Kano state, Nigeria [10], to fit the age distribution of WPV1 paralysis cases, which peaks at 2 years of age. The age distribution of cVDPV2 cases in AFRO exhibits a similar shape. This apparent protection against infection/paralysis appears to last too long to be solely attributed to maternal antibodies, and so rather than proposing other explicit biological or social mechanisms in the model, this rising susceptibility with age was incorporated to proxy the effects of maternal antibodies, reduced mixing at young ages, or other protective effects. Because this term reduces susceptibility to infection, it cannot account for any age dependence in the paralysis to infection ratio itself. Individual susceptibility vs. age is governed by the following equation:

$$\begin{aligned} \alpha\left( a \right)= \left\{ \begin{matrix} 0 {,f}_{0}+f_{1}a\leq0 \\ f_{0}+f_{1}a , 0< f_{0}+f_{1}a<1 \\ 1 {, f}_{0}+f_{1}a\geq1 \end{matrix} \right.\#\left( 1 \right) \end{aligned}$$

Where *a* is the age of the individual, and *f_0_ =* 0and *f_1_* = 0.5, so that individuals are born fully immune and become fully susceptible around 2 years of age.

Secondary infections arising from individual campaigns are not separately tracked, preventing the tracking of separate VDPV lineages undergoing independent reversion. Rather, infectivity is described as a function of time, held constant through the initial campaigns in the outbreak response and beginning to rise after the final campaign. The appropriate function to use is difficult to constrain from data. The transmission chains of unreverted, or “young” Sabin 2 viruses are very difficult to observe due to an exceedingly low case-to-infection ratio. This makes estimating the viral transmissibility itself from data difficult, and extending that to understanding how it might change as Sabin diverges from the reference strain is even more difficult. However, it appears plausible that a mutation conferring increased transmissibility would be quite beneficial and would spread quickly once it arose, so an exponential function linking the initial and final infectivity values was chosen over, e.g., a step function or linear function. The final form chosen is:

$$\begin{aligned} \beta\left( t \right)= \left\{ \begin{matrix} \begin{matrix} {g\beta}_{f} & ,t<t_{d} \end{matrix} \\ \begin{matrix} \beta_{f}*\left( 1-\left( 1-g \right)e^{\frac{t_{d}-t}{\lambda}} \right) & ,t\geq t_{d} \end{matrix} \end{matrix} \right.\#\left( 2 \right) \end{aligned}$$

Where *g* is the ratio of initial Sabin 2 infectivity to final infectivity, set to 0.25 or 0.5 in the parameter scenarios explored; $\beta_{f}$ is the final infectivity of reverted Sabin 2 virus, varied in the parameter scenarios such that the final R_0_ is 1.2, 1.5, 2, or 3 in the 0-5 cohort (see Table 1 for R_0_ calculation); *t* is the absolute simulation time in days; *t_d_* is the delay before susceptibility begins to rise (set to 86 days, the date of the final simulated response campaign); and $\lambda$ is the timescale of reversion, set to 60 or 150 days in the parameter scenarios.

Individuals of any disease state and age migrate between metapopulations (nodes), which represent AdminL1 (provinces) of 16 West African countries (Senegal, Mauritania, Sierra Leone, Guinea, Liberia, Cote d’Ivoire, Mali, Burkina Faso, Ghana, Togo, Benin, Niger, Nigeria, Cameroon, Chad, Central African Republic). The infection process in a given node and at a given timestep is governed by individuals in that node at that timestep only. Individuals migrate between nodes (from a home node *i* to destination node *j*) with relative destination node rates according to a gravity-like model of migration; migration is modeled as one-day round-trips to prevent unrealistic population accumulation in the largest nodes.

$$\begin{aligned} M{}_{ij}=\kappa\frac{p_{j}}{d_{ij}^{c}}\#\left( 3 \right) \end{aligned}$$

Where$M{}_{ij}$is the per-person, per-day probability of migration from node *i* to node *j*;$p_{j}$ is the population of node *j*; $d_{ij}$is the distance between nodes *i* and *j*; the exponent *c* in Eq. (3) is varied in the parameter scenarios, taking values *c* = 1 and *c* = 2; and the overall scale of migration is set by $\kappa$, which is varied in the separatrix runs, and sets the mean per-person per-day migration probability that is the y-axis of the Figures 1 and 3-5 in the main manuscript.

**Individual-level transitions**

With all of this in hand, individuals can be specified by a state space of {disease state *X*, age *a*, time in disease state *t_X_,* home node *i*, current node *j,* acquisition-modulating immunity α, transmission-modulating immunity τ}. The absolute simulation time *t* also plays a role due to immunization campaigns on specific dates and seasonal dynamics; this simulation state variable is shared by all individuals. Disease state transitions, vital dynamic transitions, and migration transitions are treated independently from one another, simplifying the specification of the transition space. The individual-level state transitions are presented below in the order in which they are processed in simulation.

For the sake of readability, elements of the full state$\{X, a, t_{X}, i, j, \alpha, \tau\}$ that do not change in a given transition will be suppressed. *S, E,* and *I* without subscripts will indicate an individual’s disease state, and with subscripts *j* will indicate the total population in state *X* in node *j*. *N­_j_* indicates the total population of node *j*.

The first transition in a given timestep is aging:

$$\begin{aligned} p\left( \left\{ a, t_{X} \right\}\to\left\{ a+\Delta, t_{X}+\Delta\right\} \right)=1\#\left( 4 \right) \end{aligned}$$

Next, OPV and IPV immunization interventions are processed:

| IPV immunization: | $\begin{aligned} p\left( \left\{ \alpha, \tau\right\}\to\left\{ \alpha^{*}, \tau^{*} \right\} \right)=C_{kj}\delta\left( t-t_{k} \right) \#(5)\#\# \end{aligned}$ |
| --- | --- |
| OPV immunization: | $\begin{aligned} p\left( \left\{ S, t_{S} \right\}\to\left\{ E,0 \right\} \right)=\alpha C_{kj}\delta\left( t-t_{k} \right) \#\left( 6 \right) \end{aligned}$ |

Where $C_{kj}$ indicates the effective coverage of the campaign *k* in node *j* (accounting for the effective take of OPV), $\delta$ is the Kronecker delta function, and $t_{k}$ is the date of campaign *k*.

The infectious dynamics follow:

The total infectiousness in node *j* is given by the sum over infectious individuals *m* in node *j:*

$$\begin{aligned} \beta_{tot, j}=\sum_{m=1}^{I_{j}} \tau_{m}*\beta(t)\# \end{aligned}\left( 7 \right)$$

| $\begin{aligned} p\left( \left\{ S, t_{S} \right\}\to\left\{ E, 0 \right\} \right)=1-exp \left( -\frac{\Delta\beta_{tot, j}*\alpha}{N_{j}} \right)\#\left( 8 \right) \end{aligned}$ |
| --- |
| $\begin{aligned} p\left( \left\{ E, t_{E} \right\}\to\left\{ I, 0 \right\} \right)=\delta\left( t_{E}-\gamma_{E} \right)\#\left( 9 \right) \end{aligned}$ |
| $\begin{aligned} p\left( \left\{ I, t_{I}, \alpha, \tau\right\}\to\left\{ S, 0, \alpha^{*}, \tau^{*} \right\} \right)=\delta\left( t_{I}-\gamma_{I} \right)\#\left( 10 \right) \end{aligned}$ |

That is, the exposed and infectious periods in this model are fixed values rather than distributions, so that individuals transition out of states E and I after spending exactly $\gamma_{E}$­ and $\gamma_{I}$days in the respective states. Both acquisition and transmission immunity modifiers are updated at clearance of infection. Deaths are processed next:

| $\begin{aligned} p\left( \left\{ X, a, t_{X}, i, j, \alpha, \tau\right\}\to\left\{ \emptyset\right\} \right)=\Delta\nu\left( a \right)\#\left( 11 \right) \end{aligned}$ |
| --- |

Followed by migration – again, outbound migration follows the rates in Eq. ( 2 ), and homebound migration always takes place the following timestep:

| $\begin{aligned} p\left( \left\{ i, i \right\}\to\left\{ i, j\neq i \right\} \right)={\Delta M}_{ij}\#\left( 12 \right) \end{aligned}$ |
| --- |
| $\begin{aligned} p\left( \left\{ i, j\neq i \right\}\to\left\{ i, i \right\} \right)=1\#\left( 13 \right) \end{aligned}$ |

Finally, new births are processed:

| $\begin{aligned} N\left( \left\{ \emptyset\right\}\to\left\{ S, 0, 0, i, i, 0, 0 \right\} \right)=Poiss\left( \Delta\mu N_{i}\left( t \right) \right)\#\left( 14 \right) \end{aligned}$ |
| --- |

Equations ( 1-14 ) combine to specify the agent-based model as implemented. This specification can also be converted into a set of stochastic difference equations; the resulting equations are rather unwieldy and somewhat difficult to read, limiting how informative they are to the reader. A subset of the most relevant model parameters are summarized in Table 1.

| Parameter description (symbol) | Value(s) | Relevant effect or equation | Comparison references |
| --- | --- | --- | --- |
| OPV transmission immunity modifier ($\tau_{OPV}$) | 0.9 | Upon clearance of OPV infection or IPV immunization in OPV-exposed individuals: $\tau\to\tau*(1-\tau_{OPV})$ | [1, 2, 4] |
| OPV acquisition immunity modifier ($\alpha_{OPV}$) | 0.6 | Upon clearance of OPV infection or IPV immunization in OPV-exposed individuals: $\alpha\to\alpha*(1-\alpha_{OPV})$ | [1, 2, 4] |
| IPV transmission immunity modifier ($\tau_{IPV}$) | 0.1 | Upon IPV immunization in OPV-naïve individuals: $\tau\to\tau*(1-\tau_{IPV})$ | [1, 2, 4] |
| IPV acquisition immunity modifier ($\alpha_{IPV}$) | 0.1 | Upon IPV immunization in OPV-naïve individuals: $\alpha\to\alpha*(1-\alpha_{IPV})$ | [1, 2, 4] |
| Intercept of age-dependent susceptibility function (*f_0_*) | 0.0 | $\alpha\left( a \right)= \left( f_{0}+f{}_{1}a \right)$ | [10] |
| Slope of age-dependent susceptibility function (*f_1_*) | 0.5 | $\alpha\left( a \right)= \left( f_{0}+f{}_{1}a \right)$ | [10] |
| Migration rate scalar ($\kappa$) | Varied in separatrix | $M{}_{ij}=\kappa\frac{p_{j}}{d_{ij}^{c}}$ | [11] |
| Migration rate distance dependence ($c)$ | {1, 2} | $M{}_{ij}=\kappa\frac{p_{j}}{d_{ij}^{c}}$ | [11] |
| Duration of exposed state ($\gamma_{E})$ | 3 | $p\left( \left\{ E, t_{E} \right\}\to\left\{ I, 0 \right\} \right)=\delta\left( t_{E}-\gamma_{E} \right)$ | [1, 2] |
| Duration of infectious state ($\gamma_{I})$ | 27 | $p\left( \left\{ I, t_{I}, \alpha, \tau\right\}\to\left\{ S, 0, \alpha^{*}, \tau^{*} \right\} \right)=\delta\left( t_{I}-\gamma_{I}) \right)$ | [1, 2] |
| Initial infectiousness of Sabin 2 relative to fully-reverted ($g)$ | {0.25, 0.5} | $\beta\left( t \right)= \left\{ \begin{matrix} \begin{matrix} {g\beta}_{f} & ,t<t_{d} \end{matrix} \\ \begin{matrix} \beta_{f}*\left( 1-\left( 1-g \right)e^{\frac{t-t_{d}}{\lambda}} \right) & ,t\geq t_{d} \end{matrix} \end{matrix} \right.$ | [1, 12, 13] |
| Final R­_0_ of reverted Sabin 2 (*R_0f_*) | {1.2, 1.5, 2, 3} | $R_{0f}=\frac{\beta_{f}*\gamma}{\int\alpha\left( a \right) * f\left( a \right)da}$  Denominator indicates correction for age-dependent susceptibility, with $\alpha\left( a \right)$ defined as in (1) and $f\left( a \right)$ the population age distribution. |  |
| Delay before Sabin reversion ($t_{d})$ | 86 (last response campaign) | $\beta\left( t \right)= \left\{ \begin{matrix} \begin{matrix} {g\beta}_{f} & ,t<t_{d} \end{matrix} \\ \begin{matrix} \beta_{f}*\left( 1-\left( 1-g \right)e^{\frac{t-t_{d}}{\lambda}} \right) & ,t\geq t_{d} \end{matrix} \end{matrix} \right.$ |  |
| Timescale of Sabin reversion ($\lambda)$ | {60, 150} | $\beta\left( t \right)= \left\{ \begin{matrix} \begin{matrix} {g\beta}_{f} & ,t<t_{d} \end{matrix} \\ \begin{matrix} \beta_{f}*\left( 1-\left( 1-g \right)e^{\frac{t-t_{d}}{\lambda}} \right) & ,t\geq t_{d} \end{matrix} \end{matrix} \right.$ | [14] |

Table 1: Description of a set of model parameters relevant to the study

**Calibration**

In 2008, a seasonal peak in endemic transmission in Nigeria apparently seeded outbreaks that propagated over the following two years throughout the West African countries modeled in this work. Comparing this historical outbreak with the behavior of modeled outbreaks allows for qualitative bounds to be placed on the magnitude of migration rates in the model. Figure 1 presents this historical outbreak, with countries placed into a rough ordering from northwest at the top to southeast at the bottom, producing a striking traveling epidemic pattern.


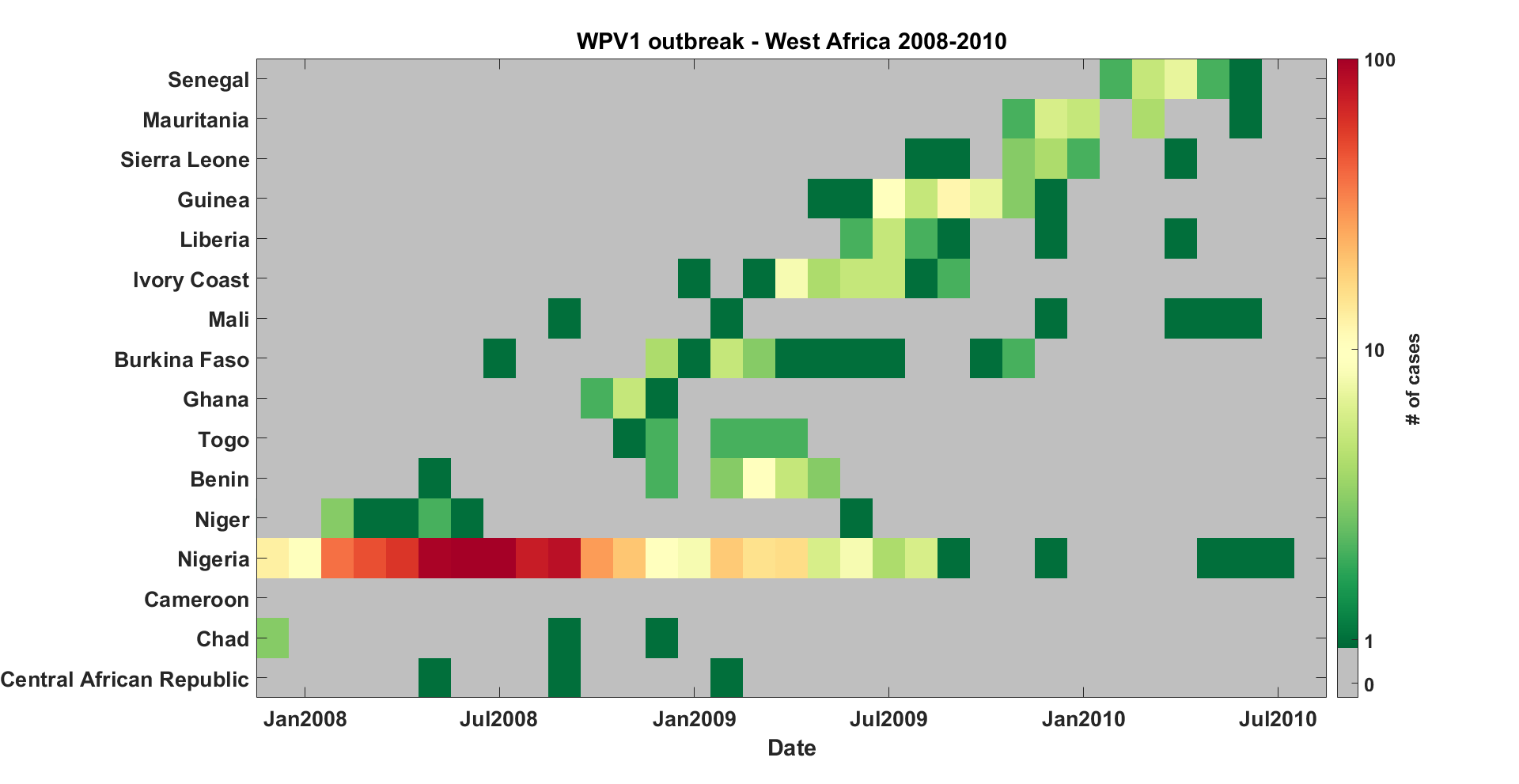


Figure 1: Wild Poliovirus Type 1 outbreak in West Africa, 2008-2010. Countries are roughly ordered northwest to southeast from top to bottom. This data was obtained from WHO’s Polio Information System (POLIS) AFP case reporting database. Permission for access to the POLIS AFP case database was granted to the Institute for Disease Modeling researchers through the World Health Organization.

Calibrating the properties of the modeled migration network to this past poliovirus outbreak presents inherent difficulties – due to the case-to-infection ratio, the spread of infection is poorly sampled; disease propagation on the network is stochastic, and only a single observation of propagation on the network is observed; and the propagation process depends not only on the structure of the migration network, but also on the uncertain transmissibility and immunity conditions in the nodes of the network. For these reasons, a detailed calibration of the migration models to the full spatial infection traces is not attempted. Rather, calibration qualitatively targets the temporal behavior of the outbreak, seeking to minimize the differences between a “case-weighted time” in each country from data (Eq. 15) and “incidence-weighted time” from simulation in each country (Eq. 16). The relative amplitudes of the outbreaks in different countries are not targeted as part of the calibration.

| $\overline{t_{j, data}}=\frac{\left( \sum_{t} t*O_{j,t} \right)}{\sum_{t} \left( O_{j, t} \right)}$ | $\left( \boldsymbol{15} \right)$ |
| --- | --- |
| $\overline{t_{j, sim}}=\frac{\left( \sum_{t} t*I_{j,t} \right)}{\sum_{t} \left( I_{j, t} \right)}$ | $\left( \boldsymbol{16} \right)$ |
| $\sigma= \sqrt{\sum_{j} \left( \overline{t_{j, data}}- \overline{t_{j, sim}} \right)^{2}}$ | $\left( \boldsymbol{17} \right)$ |

With *t* stepping in 30-day increments beginning on Jan 1, 2008; $O_{j,t}$ indicating the number of cases in country *j* during time bin *t*, and $I_{j,t}$ indicating the number of infections in country *j* during time bin *t*. As the exact peak of the Nigeria outbreak may shift from simulation to simulation, $\overline{t_{j, sim}}$ is defined relative to that peak in each simulation. The calibration aims to minimize the term $\sigma$ defined in Eq. 17 above (in practice, it actually aims to maximize $-log10(\sigma)$).

Incremental Mixture Importance Sampling (IMIS) is used to sample and re-sample a 2D parameter space of R_0_ and overall migration rate.[15] IMIS was designed to work with deterministic models and proportionally sample a true posterior density based on an appropriately-defined likelihood function. However, the author has found it effective as a means of maximizing and mapping the surface of an objective function (not necessarily likelihoods) applied to a stochastic model, so long as the stochastic variance in the objective function at a single point is outweighed by the “parameter-based” variation throughout the calibration space - in some sense, as long the objective surface is “smooth”, or has a high signal-to-noise. Additionally, a code base for interfacing IMIS directly to IDM’s computational cluster already exists from previous work.

The model utilized in calibration is not strictly equivalent to that used in the investigations of post-cessation outbreak behavior. The immunity levels in each province are not set equal to each other, but are rather derived from vaccine dose histories reported by acute flaccid paralysis cases during the time period in question.[16] Partial immunity is also not treated in the calibration version of the model; transmission takes place only through fully susceptible children. Simulations begin in January 2008, with an outbreak seeded in Nigeria, and run through June 2013 (though the comparison only considers times through July 2010).

Figure 2 presents the results of the calibration; points in the 2D space represent a single sampled pair of infectivity and $\kappa$ values, and the color indicating $-log10(\sigma)$. A broad maximum is apparent around mean migration rates from approximately -3.5 to -2.5 (in log-10 units), representing the preferred region outlined in the figures in the main text; this corresponds to the average child traveling outside of their home state approximately once per year to once per decade. Figure 3 presents the infection traces from two representative simulations in the preferred region under the actual outbreak. Above this range of migration rates, transmission across the region becomes increasingly synchronous, qualitatively ruling out these values as realistic; an example is shown in Figure 4. Below this preferred region, the metapopulations become more disconnected and transmission fails to export broadly across the network; an example is shown in Figure 5.


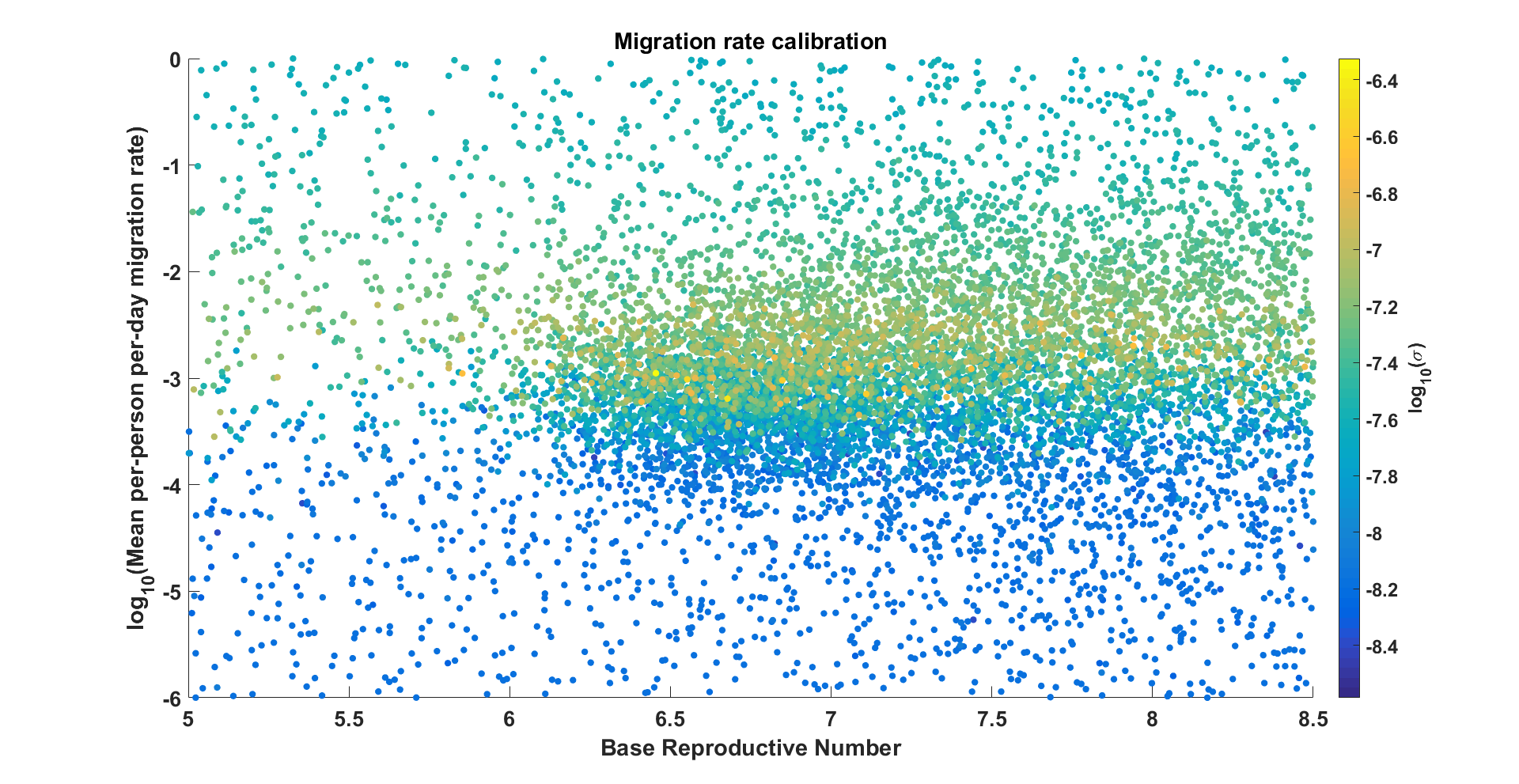


Figure 2: Results of calibration of migration rates. The x and y- axes represent the infectivity and mean migration rate, respectively, with color indicating the value of the objective function defined in Eqs (15-17). The results indicate a broad maximum around mean migration rates from roughly -3.5 to -2.5 (in log10 units), i.e., the average child leaving their home state approximately once per year to once per decade.


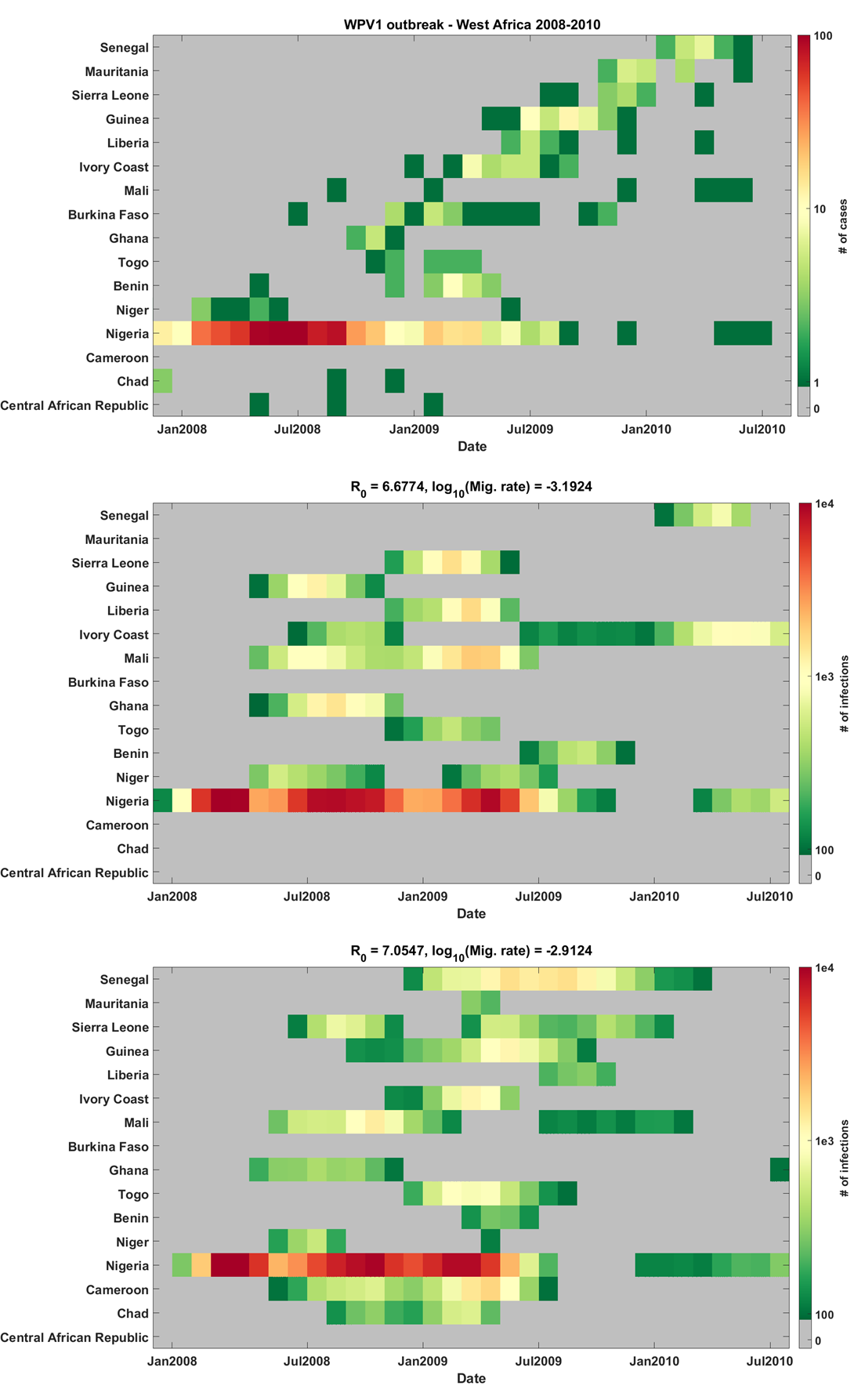


Figure 3: Top: WPV1 outbreak in West Africa, 2008-2010, representing the calibration target for this exercise. Middle and bottom: Two example simulation outputs from the preferred region of parameter space. The case data presented in the top panel was obtained from the POLIS AFP case reporting database. Permission for access to the POLIS AFP case database was granted to the Institute for Disease Modeling researchers through the World Health Organization.


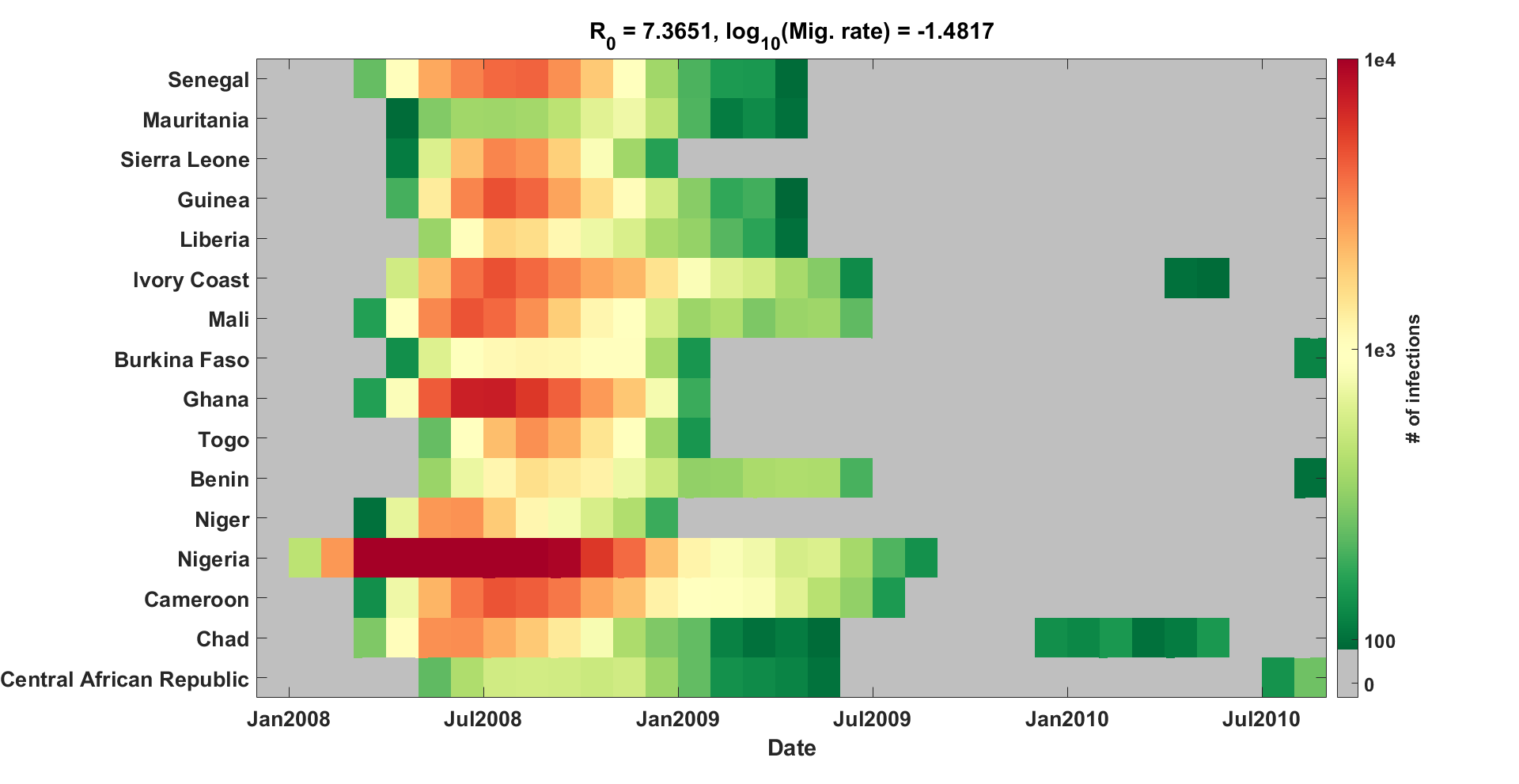


Figure 4: Example simulation output with migration rate set above the preferred range from calibration. As can be seen, transmission across the region is essentially synchronous, in contrast to the travelling outbreak actually observed.


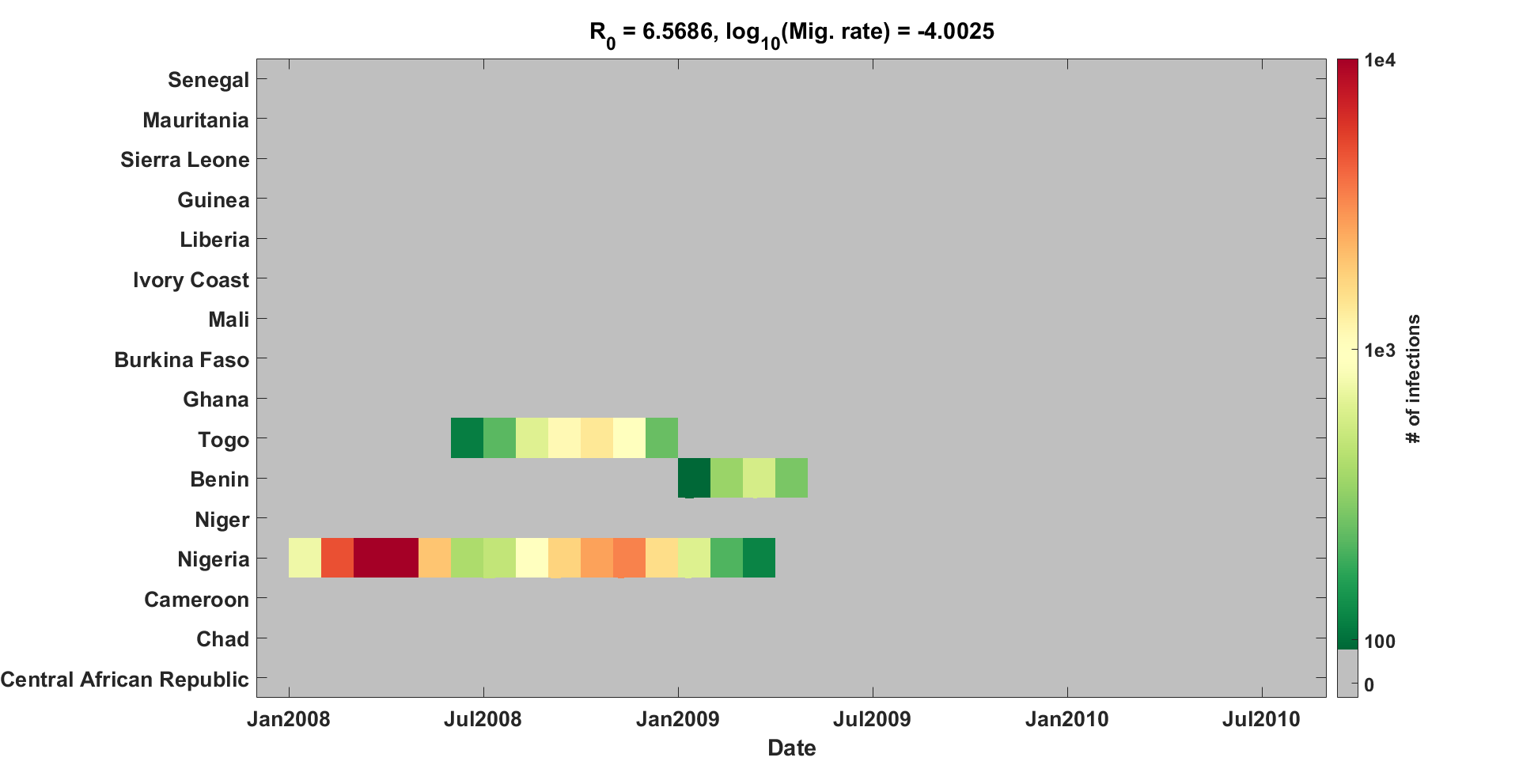


Figure 5: Example simulation output with migration rate set below the preferred range from calibration. In this case, transmission fails to export broadly across the region (though one or two exportations are not uncommon to observe), in contrast to the travelling outbreak observed in 2008.

While the simulations investigating the risk of Sabin 2 use post-cessation are allowed to explore a wide range of migration rates, this calibration allows for the identification of a preferred region of migration space, aiding interpretability of the results.

**Construction of the simulated metapopulation network**

The simulation metapopulations are generated by applying WHO shapefiles from POLIS to population maps of Africa obtained from the Worldpop collaboration.[9, 17] Figure 6 illustrates the population map (color) and country/province boundaries (thick/thin black solid lines) that were used. The total size of a given metapopulation is the aggregated sum of the per-pixel population map within the boundaries, and each population is placed at the population-weighted centroid of the province (for purposes of computing distances between metapopulations in the gravity model of migration).


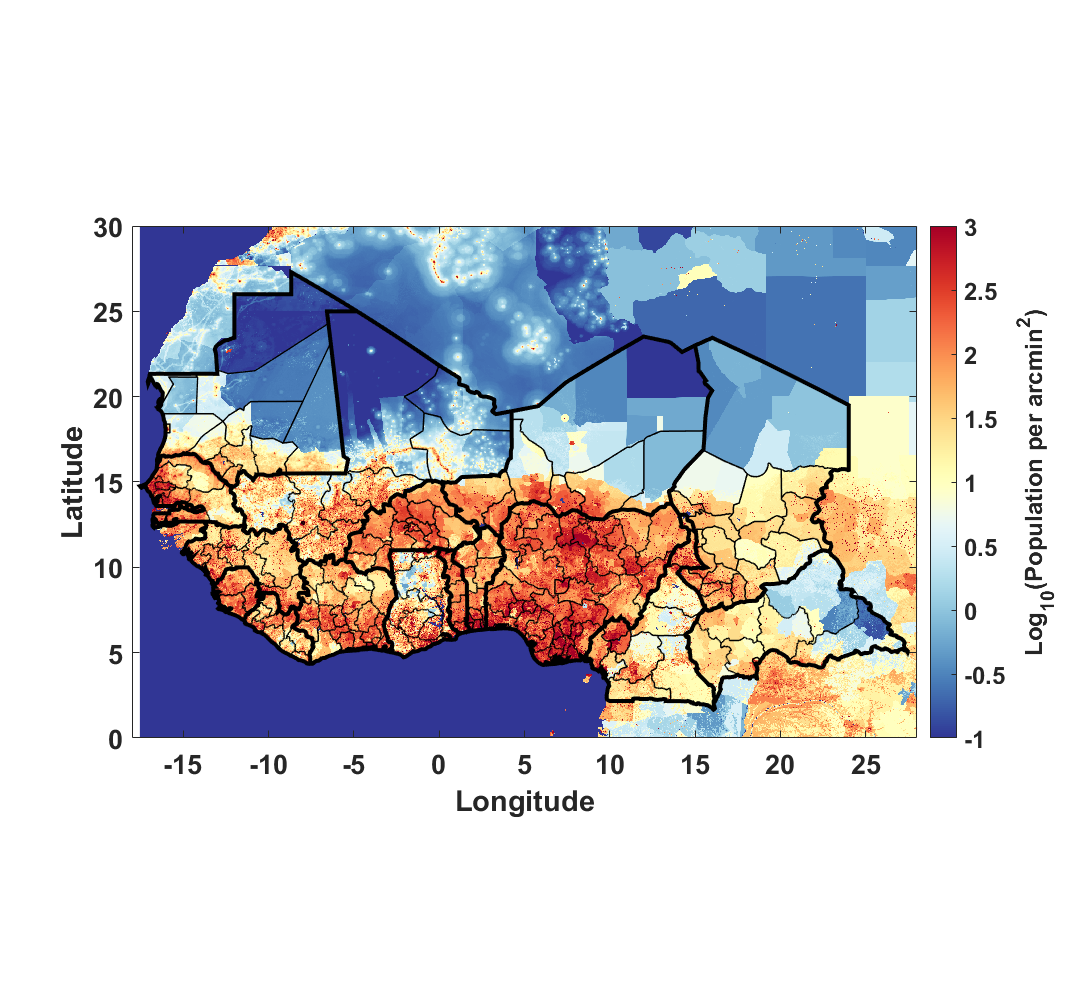


Figure 6: Population map (from Worldpop) and country/province boundaries (thick/thin solid black lines, from POLIS) used to generate the metapopulations for the simulation [9, 17]. Permission for use of the depicted boundary shapefiles was granted to the Institute for Disease Modeling researchers through the World Health Organization. The boundaries and names shown and the designations used in this map do not imply the expression of any opinion whatsoever on the part of the World Health Organization concerning the legal status of any country, territory, city, or area or of its authorities, or concerning the delimitation of its frontiers or boundaries.

**Additional scenario comparison – Inverse-linear vs. inverse-square migration model**

Changing the distance-dependence in the gravity model of migration is found to have a negligible effect on the position of the separatrix line, with an inverse-linear distance-dependence (*c*=1, relatively higher migration rates to provinces of highest population) producing very slightly more risk than an inverse-square dependence (*c*=2, relatively higher migration rates to nearest neighbor provinces). Figure 7 presents the results of this comparison. In general, at equal total mean migration rate, the inverse-square migration model favoring more nearest-neighbor type migration is slightly less likely to have established exportation than the inverse-linear model, favoring long-distance migration to population centers. However, the effect is quite small; the overall connectedness of the metapopulation network (i.e., the y-axis of the figure) is more important than the distinction between these two schemes for the relative weights of the connections.


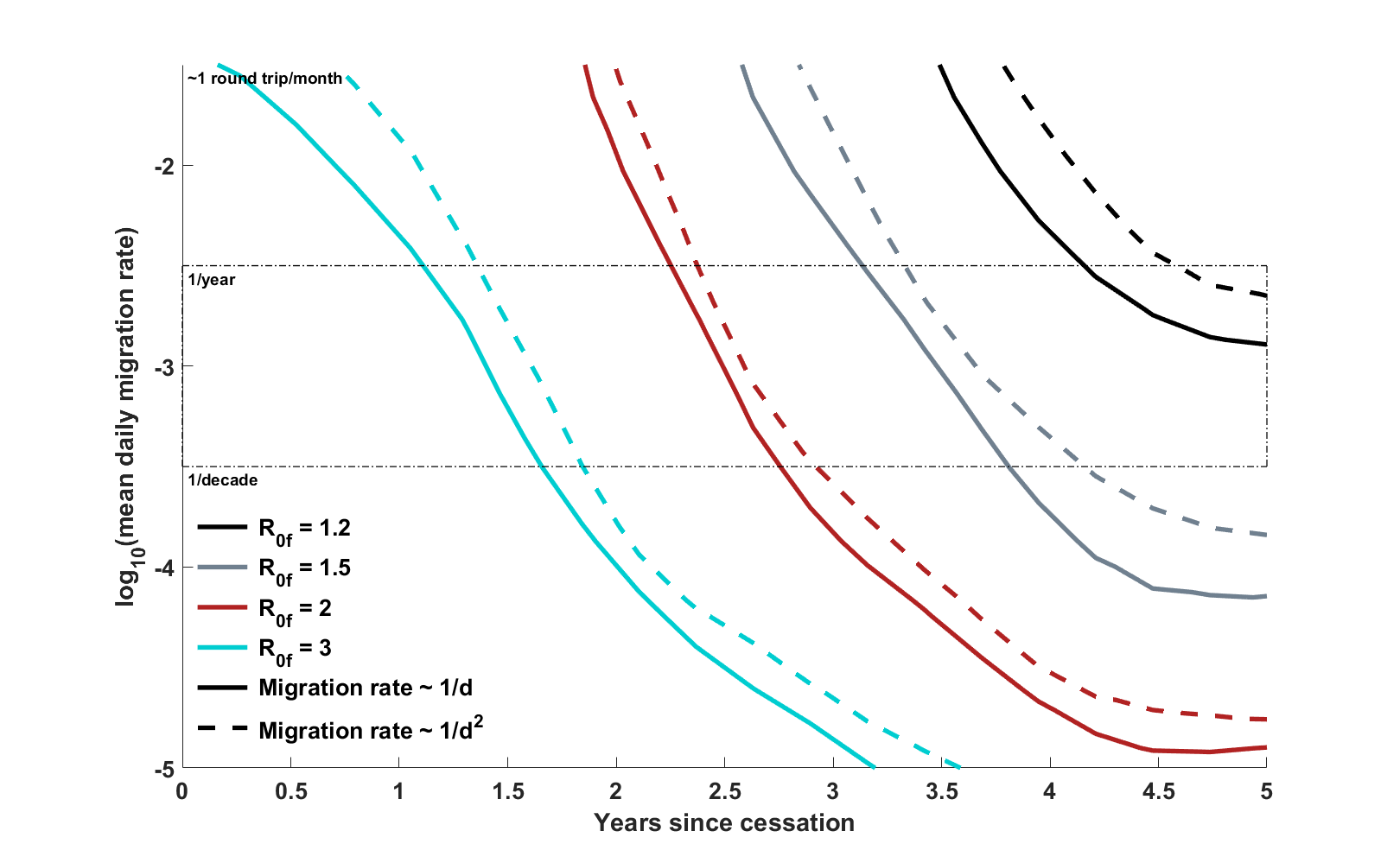


Figure 7: Position of the 50% separatrix line as the distance dependence in the gravity model of migration varies, at constant *λ*= 60 days, N­_IPV_= 1, *c*= 1. The solid and dashed lines respectively indicate c = 1 and c = 2, while the cyan, red, grey, and black respectively indicate R_0f_ values of 3, 2, 1.5, and 1.2. The relative distribution of migration rates in the network induces a negligible effect compared with the overall mean migration rate of individuals on the network to all possible destinations.

**Additional scenario comparison – Timescale of reversion**

Figure 8 shows that increasing the timescale of Sabin 2 reversion from *λ*= 60 days to *λ*= 150 days provides a moderate mitigation of the survival risk, similar to the effect of changing *f* from 0.5 to 0.25 and small compared to the effects of changing R_0f_ or N_IPV_. The use of an exponential reversion function, in which the recovery of transmissibility occurs most quickly early in the reversion process, could be one reason for this. A second consideration is that even at very low values of the effective reproduction rate, population prevalence is exceedingly high immediately following a campaign – in the context of a homogenously mixed model, this will allow a few generations of transmission even at low R_0_, enabling the virus to recover transmissibility. In this situation, the overall population immunity and its relation to the final R_0_ would dominate the probability of the OPV2 survival outcome, with the reversion rate playing only a secondary role. This line of reasoning is supported by the observation, from Figure 8, that a longer reversion timescale has a greater impact at the lower values of R_0f_ tested in these scenarios, as the effective reproductive number of the circulating Sabin 2 remains below 1 for multiple generations of infection after the campaigns.


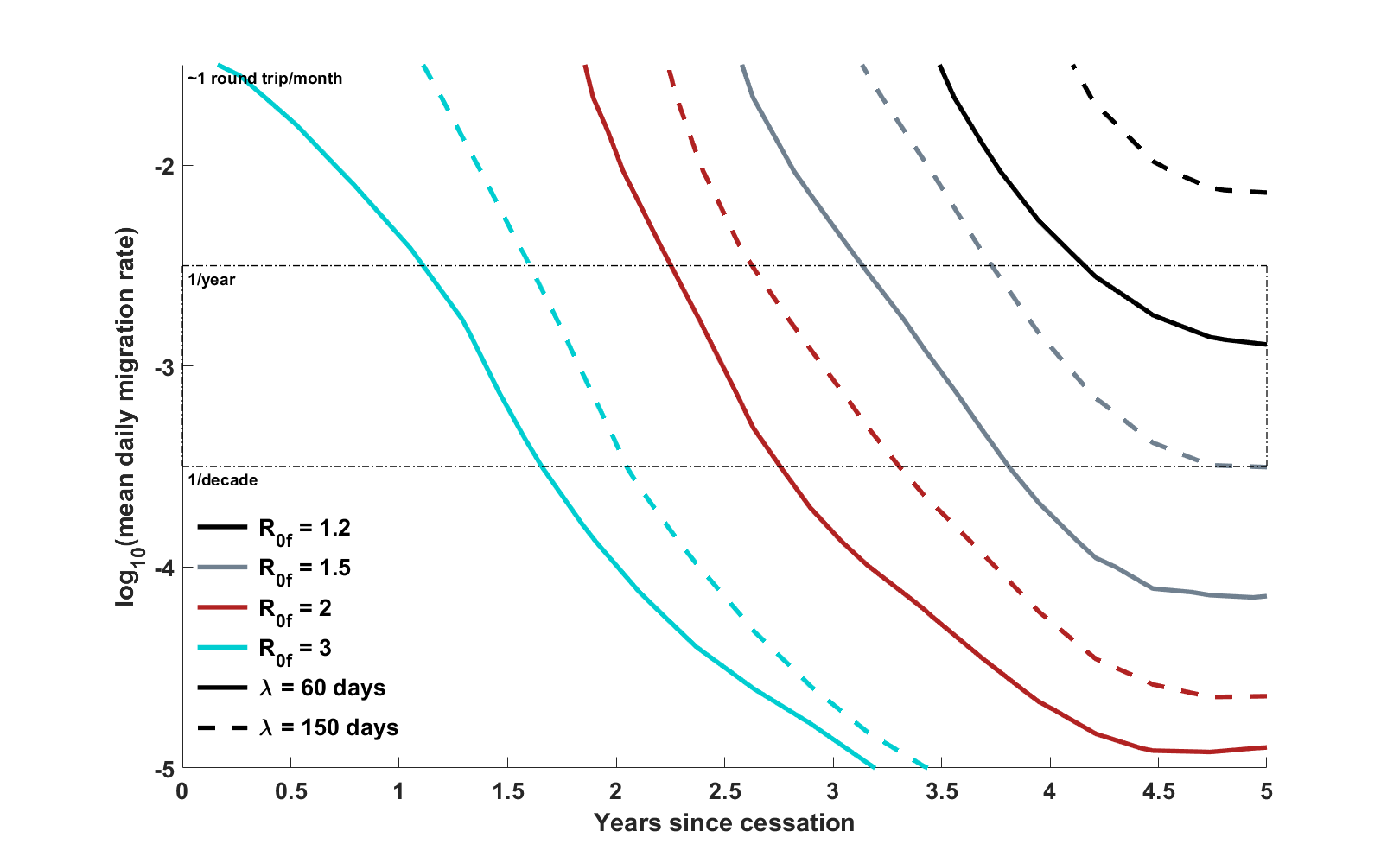


Figure 8: Position of the 50% separatrix line as the timescale of R_0_ reversion varies, at constant f=0.5, N­_IPV_= 1, *c*= 1. The solid and dashed lines respectively indicate *λ*= 60 days and *λ*= 150 days, while the cyan, red, grey, and black respectively indicate R_0f_ values of 3, 2, 1.5, and 1.2. The final R_0_ is observed to have the dominant effect. At the higher values of R_0f_, changing the reversion timescale provides minimal mitigation of the Sabin 2 survival risk, but the effect grows at lower values of R_0f_, as the effective reproductive rate of Sabin 2 remains below 1 for more transmission generations.

**Is the 9 month endpoint for OPV2 survival long enough?**

Figures 9 and 10 represent the results of a check that 9 months of circulation after the outbreak response is a reasonable endpoint for considering Sabin 2 to have survived to create a new cVDPV2. Both panels of Figure 9 present results from running the Separatrix algorithm given the same parameter scenario (R_0f_ = 2.0, g = 0.5, *λ*= 60 days, N­_IPV_= 1, *c*= 1). Sabin 2 to have successfully established a new lineage given 9 months of circulation after the response in the left panel (the criterion used in the results throughout the main manuscript), but after 30 months of persistent circulation in the right panel. The results are quite similar in the two panels. Figure 10 specifically compares the 50% Separatrix lines from the two runs to more clearly illustrate the similarity in the results. These figures demonstrate that in this scenario, observing persistent circulation for 9 months is sufficient to demonstrate establishment of a viral lineage that will survive long-term. While this is only a single scenario, and this spot-check does not guarantee similar results for all scenarios, it serves to provide a degree of support to the validity of using the 9-month endpoint throughout the manuscript.

Figure 9: Comparison of Separatrix outputs, both with R_0f_ = 2.0, g = 0.5, *λ*= 60 days, N­_IPV_= 1, *c*= 1. On the left, the Sabin 2 is required to survive for 9 months outside of the response region after the final campaign; on the right, it must survive for 30 months to be considered a surviving VDPV lineage. The colored surfaces represents the inferred probability of OPV2 survival, and the black solid lines represents the parameter contour along which this survival probability is 50%. Gray crosses represent individual simulations in OPV2 survives, and gray circles represent those in which it does not.


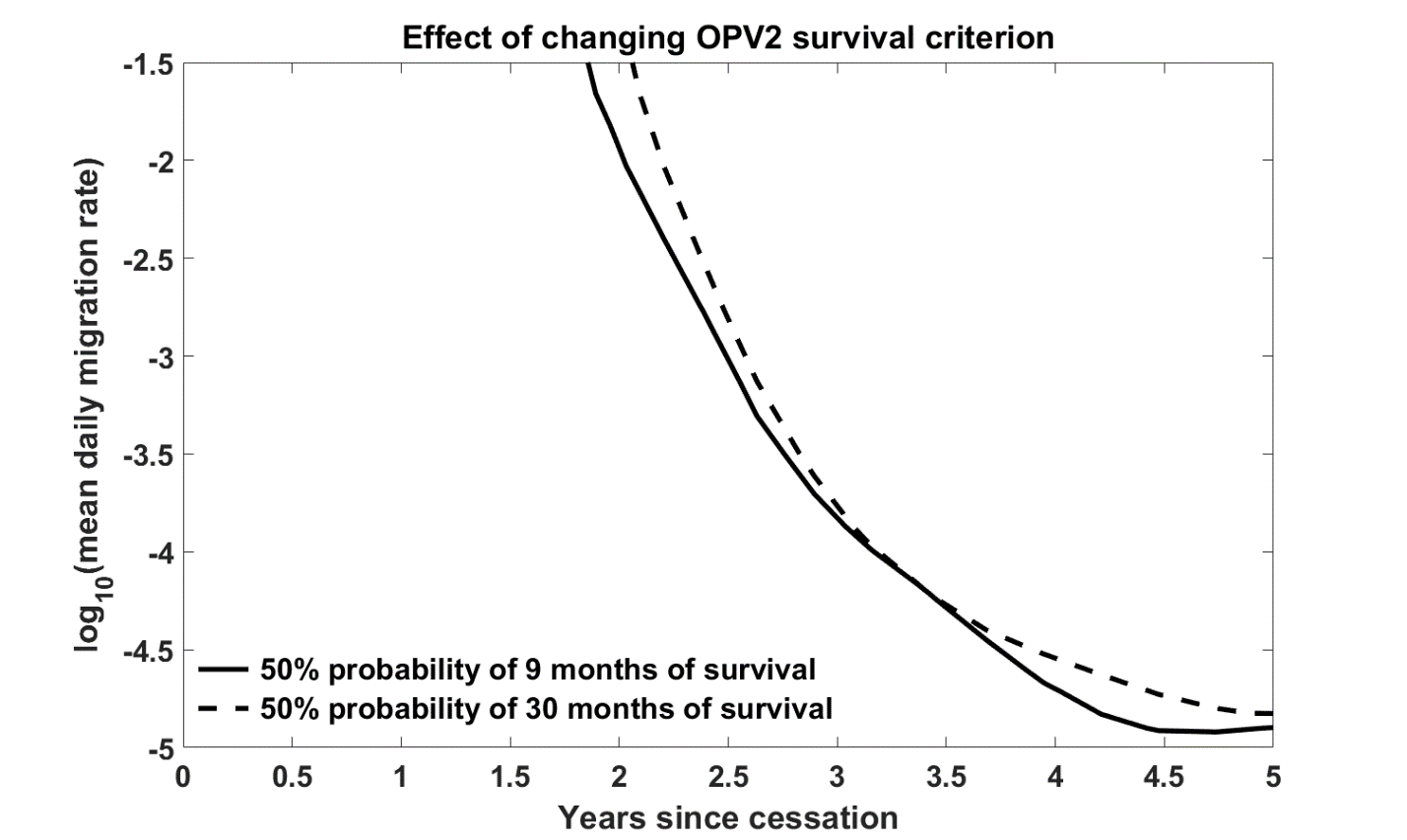


Figure 10: Comparison of the 50% separatrix lines from Figure 9 left (solid) and right (dashed), demonstrating that the results are not substantially changed by extending the criterion for successful Sabin 2 survival from 9 months of circulation to 30 months of circulation. These results were spot-checked for a single parameter scenario, with R_0f_ = 2.0, g = 0.5, *λ*= 60 days, N­_IPV_= 1, *c*= 1, and does not guarantee that the results are similar in every single parameter scenario.

**References**

1. Duintjer Tebbens RJ, Pallansch MA, Kalkowska DA, Wassilak SGF, Cochi SL, Thompson KM. Characterizing poliovirus transmission and evolution: insights from modeling experiences with wild and vaccine-related polioviruses. Risk Anal. 2013;33:703–49. doi:10.1111/risa.12044.

2. Behrend MR, Hu H, Nigmatulina KR, Eckhoff P. A quantitative survey of the literature on poliovirus infection and immunity. Int J Infect Dis. 2014;18:4–13.

3. Duintjer Tebbens RJ, Pallansch MA, Chumakov KM, Halsey NA, Hovi T, Minor PD, et al. Expert Review on Poliovirus Immunity and Transmission. Risk Anal. 2013;33:544–605. doi:10.1111/j.1539-6924.2012.01864.x.

4. Hird TR, Grassly NC. Systematic review of mucosal immunity induced by oral and inactivated poliovirus vaccines against virus shedding following oral poliovirus challenge. PLoS Pathog. 2012;8:e1002599. doi:10.1371/journal.ppat.1002599.

5. Famulare M, Chabot-Couture G, Eckhoff PA, Lyons H, McCarthy KA, Selinger C. How polio vaccination affects poliovirus transmission. bioRxiv. 2016. http://biorxiv.org/content/early/2016/10/27/084012. Accessed 20 May 2017.

6. O’Ryan M, Bandyopadhyay AS, Villena R, Espinoza M, Novoa J, Weldon WC, et al. Inactivated poliovirus vaccine given alone or in a sequential schedule with bivalent oral poliovirus vaccine in Chilean infants: a randomised, controlled, open-label, phase 4, non-inferiority study. Lancet Infect Dis. 2015;15:1273–82. doi:10.1016/S1473-3099(15)00219-4.

7. Asturias EJ, Bandyopadhyay AS, Self S, Rivera L, Saez-Llorens X, Lopez E, et al. Humoral and intestinal immunity induced by new schedules of bivalent oral poliovirus vaccine and one or two doses of inactivated poliovirus vaccine in Latin American infants: an open-label randomised controlled trial. Lancet. 2016;388:158–69. doi:10.1016/S0140-6736(16)00703-0.

8. National Population Commission - Federal Republic of Nigeria, ICF International. Nigeria Demographic and Health Survey, 2013 - Final Report. 2014.

9. POLIS: The polio information system. https://extranet.who.int/polis/Search. Accessed 2 Aug 2016.

10. McCarthy KA, Chabot-Couture G, Shuaib F. A spatial model of Wild Poliovirus Type 1 in Kano State, Nigeria: calibration and assessment of elimination probability. BMC Infect Dis. 2016;16. doi:10.1186/s12879-016-18.

11. Wesolowski A, Buckee CO, Pindolia DK, Eagle N, Smith DL, Garcia AJ, et al. The Use of Census Migration Data to Approximate Human Movement Patterns across Temporal Scales. PLoS One. 2013;8:e52971. doi:10.1371/journal.pone.0052971.

12. Eichner M, Dietz K. Eradication of poliomyelitis: when can one be sure that polio virus transmission has been terminated? Am J Epidemiol. 1996;143:816–22. http://www.ncbi.nlm.nih.gov/pubmed/8610692. Accessed 14 Aug 2015.

13. Pons-Salort M, Burns CC, Lyons H, Blake IM, Jafari H, Oberste MS, et al. Preventing Vaccine-Derived Poliovirus Emergence during the Polio Endgame. PLOS Pathog. 2016;12:e1005728. doi:10.1371/journal.ppat.1005728.

14. Duintjer Tebbens RJ, Pallansch MA, Kim J-H, Burns CC, Kew OM, Oberste MS, et al. Oral poliovirus vaccine evolution and insights relevant to modeling the risks of circulating vaccine-derived polioviruses (cVDPVs). Risk Anal. 2013;33:680–702. doi:10.1111/risa.12022.

15. Raftery AE, Bao L. Estimating and Projecting Trends in HIV/AIDS Generalized Epidemics Using Incremental Mixture Importance Sampling. Biometrics. 2010;66:1162–73. doi:10.1111/j.1541-0420.2010.01399.x.

16. Upfill-Brown AM, Lyons HM, Pate MA, Shuaib F, Baig S, Hu H, et al. Predictive spatial risk model of poliovirus to aid prioritization and hasten eradication in Nigeria. BMC Med. 2014;12:92. doi:10.1186/1741-7015-12-92.

17. WorldPop Collaboration. Worldpop - Selected Data: Africa &gt; Whole Continent &gt; Population 2010. 2012. http://www.worldpop.org.uk/data/summary/?contselect=Africa&countselect=Whole+Continent&typeselect=Population+2010. Accessed 24 Aug 2016.
