# Supplementary material for: The risk of type 2 oral polio vaccine use in post-cessation outbreak response
Source: BMC Med. 2017 Oct 4;15:175. doi: 10.1186/s12916-017-0937-y (PMC5627419; doi:10.1186/s12916-017-0937-y)
Supplement: Additional file 1: — Supplemental Materials. (ZIP 2.67 mb) [file 12916_2017_937_MOESM1_ESM.zip › Supplemental Table of Symbols.docx]

Table of all symbols used in Supplement equations, in order of appearance.

| Symbol | Equations of appearance | Interpretation |
| --- | --- | --- |
| α | (1), (5), (6), (10), (11) | Acquisition-moderating immunity. 1 = naïve, 0 = immune |
| *a* | (1), (4), (11) | Age of individual |
| *f*_0_ | (1) | Individual susceptibility to acquisition at birth |
| *f*_1_ | (1) | Rise in susceptibility with age |
| $\beta\left( t \right)$ | (2) | Daily infectivity of single individual, as a function of time |
| *g* | (2) | Ratio of initial Sabin infectivity to that of fully reverted Sabin 2. |
| $\beta_{f}$ | (2) | Final infectivity of fully reverted Sabin 2 |
| *t* | (2) | Simulation time in days |
| *t_d_* | (2) | Delay before Sabin infectivity begins to rise |
| $\lambda$ | (2) | Timescale of reversion of Sabin 2 infectivity |
| $M{}_{ij}$ | (3), (12) | Per-person, per-day probability of migration from *i* to *j* |
| *i* | (3), (12), (13) | Individual’s home node (when not migrated) |
| *j* | (3), (12), (13) | Individual’s current node (during migration) |
| $\kappa$ | (3) | Multiplicative scalar on the inter-node migration matrix |
| $p_{j}$ | (3) | Population of node *j* |
| $d_{ij}$ | (3) | Distance between nodes *i* and *j* |
| *c* | (3) | Exponent on inter-node distance in migration rates |
| $t_{X}$ | (4), (6), (9), (10), (11) | Individual time spent in disease state X, where X = {S, E, I} |
| *X* | (4), (11) | Indicator for individual disease state, where X = {S, E, I} |
| $\Delta$ | (4), (8), (11), (14) | Simulation timestep, in days |
| $\delta$ | (5), (6), (9), (10) | Kronecker delta function |
| $\tau$ | (5), (7), (10) | Transmission-moderating immunity. 1 = naïve, 0 = immune |
| $C_{kj}$ | (5), (6) | Coverage of campaign *k* in node *j* |
| $t_{k}$ | (5), (6) | Date of campaign *k* |
| $\beta_{tot, j}$ | (7), (8) | Total infectivity shed by all infectious individuals in node *j* |
| $N_{j}$ | (8), (14) | Number of individuals in node *j* |
| $\gamma_{E}$ | (9) | Duration of exposed period |
| $\gamma_{I}$ | (10) | Duration of infectious period |
| $\nu\left( a \right)$ | (11) | Age-specific mortality rate |
| $\mu$ | (14) | Birth rate |
| $\overline{t_{j, data}}$ | (15) | Case-weighted outbreak time in country *j* from the 2008-2010 West Africa WPV1 outbreak*,* used to fit migration model |
| $O_{j,t}$ | (15) | Observed cases in country *j* in month *t* |
| $\overline{t_{j, sim}}$ | (15) | Incidence-weighted outbreak time in country *j* from simulations of the 2008-2010 West Africa WPV1 outbreak*,* used to fit migration model |
| $I_{j,t}$ | (15) | Simulated incidence in country *j* in month *t* |
| $\sigma$ | (17) | Square root of the sum, over countries, of squared differences between case-weighted outbreak times from data and incidence-weighted outbreak times from simulation, for the 2008-2010 WPV1 outbreak in West Africa |

Table of particularly relevant parameters, with corresponding values, equations, and references to other models for comparison.

| Parameter description (symbol) | Value(s) | Relevant effect or equation | Comparison references |
| --- | --- | --- | --- |
| OPV transmission immunity modifier ($\tau_{OPV}$) | 0.9 | Upon clearance of OPV infection or IPV immunization in OPV-exposed individuals: $\tau\to\tau*(1-\tau_{OPV})$ | [1, 2, 4] |
| OPV acquisition immunity modifier ($\alpha_{OPV}$) | 0.6 | Upon clearance of OPV infection or IPV immunization in OPV-exposed individuals: $\alpha\to\alpha*(1-\alpha_{OPV})$ | [1, 2, 4] |
| IPV transmission immunity modifier ($\tau_{IPV}$) | 0.1 | Upon IPV immunization in OPV-naïve individuals: $\tau\to\tau*(1-\tau_{IPV})$ | [1, 2, 4] |
| IPV acquisition immunity modifier ($\alpha_{IPV}$) | 0.1 | Upon IPV immunization in OPV-naïve individuals: $\alpha\to\alpha*(1-\alpha_{IPV})$ | [1, 2, 4] |
| Intercept of age-dependent susceptibility function (*f_0_*) | 0.0 | $\alpha\left( a \right)= \left( f_{0}+f{}_{1}a \right)$ | [10] |
| Slope of age-dependent susceptibility function (*f_1_*) | 0.5 | $\alpha\left( a \right)= \left( f_{0}+f{}_{1}a \right)$ | [10] |
| Migration rate scalar ($\kappa$) | Varied in separatrix | $M{}_{ij}=\kappa\frac{p_{j}}{d_{ij}^{c}}$ | [11] |
| Migration rate distance dependence ($c)$ | {1, 2} | $M{}_{ij}=\kappa\frac{p_{j}}{d_{ij}^{c}}$ | [11] |
| Duration of exposed state ($\gamma_{E})$ | 3 | $p\left( \left\{ E, t_{E} \right\}\to\left\{ I, 0 \right\} \right)=\delta\left( t_{E}-\gamma_{E} \right)$ | [1, 2] |
| Duration of infectious state ($\gamma_{I})$ | 27 | $p\left( \left\{ I, t_{I}, \alpha, \tau\right\}\to\left\{ S, 0, \alpha^{*}, \tau^{*} \right\} \right)=\delta\left( t_{I}-\gamma_{I}) \right)$ | [1, 2] |
| Initial infectiousness of Sabin 2 relative to fully-reverted ($g)$ | {0.25, 0.5} | $\beta\left( t \right)= \left\{ \begin{matrix} \begin{matrix} {g\beta}_{f} & ,t<t_{d} \end{matrix} \\ \begin{matrix} \beta_{f}*\left( 1-\left( 1-g \right)e^{\frac{t-t_{d}}{\lambda}} \right) & ,t\geq t_{d} \end{matrix} \end{matrix} \right.$ | [1, 12, 13] |
| Final R­_0_ of reverted Sabin 2 (*R_0f_*) | {1.2, 1.5, 2, 3} | $R_{0f}=\frac{\beta_{f}*\gamma}{\int\alpha\left( a \right) * f\left( a \right)da}$  Denominator indicates correction for age-dependent susceptibility, with $\alpha\left( a \right)$ defined as in (1) and $f\left( a \right)$ the population age distribution. |  |
| Delay before Sabin reversion ($t_{d})$ | 86 (last response campaign) | $\beta\left( t \right)= \left\{ \begin{matrix} \begin{matrix} {g\beta}_{f} & ,t<t_{d} \end{matrix} \\ \begin{matrix} \beta_{f}*\left( 1-\left( 1-g \right)e^{\frac{t-t_{d}}{\lambda}} \right) & ,t\geq t_{d} \end{matrix} \end{matrix} \right.$ |  |
| Timescale of Sabin reversion ($\lambda)$ | {60, 150} | $\beta\left( t \right)= \left\{ \begin{matrix} \begin{matrix} {g\beta}_{f} & ,t<t_{d} \end{matrix} \\ \begin{matrix} \beta_{f}*\left( 1-\left( 1-g \right)e^{\frac{t-t_{d}}{\lambda}} \right) & ,t\geq t_{d} \end{matrix} \end{matrix} \right.$ | [14] |
